# Dynamic Chromatin Organization during Foregut Development Mediated by the Organ Selector Gene PHA-4/FoxA

**DOI:** 10.1371/journal.pgen.1001060

**Published:** 2010-08-12

**Authors:** Tala H. I. Fakhouri, Jeff Stevenson, Andrew D. Chisholm, Susan E. Mango

**Affiliations:** Department of Molecular and Cellular Biology, Harvard University, Cambridge, Massachusetts, United States of America; Stanford University School of Medicine, United States of America

## Abstract

Central regulators of cell fate, or selector genes, establish the identity of cells by direct regulation of large cohorts of genes. In *Caenorhabditis elegans*, foregut (or pharynx) identity relies on the FoxA transcription factor PHA-4, which activates different sets of target genes at various times and in diverse cellular environments. An outstanding question is how PHA-4 distinguishes between target genes for appropriate transcriptional control. We have used the Nuclear Spot Assay and GFP reporters to examine PHA-4 interactions with target promoters in living embryos and with single cell resolution. While PHA-4 was found throughout the digestive tract, binding and activation of pharyngeally expressed promoters was restricted to a subset of pharyngeal cells and excluded from the intestine. An RNAi screen of candidate nuclear factors identified emerin (*emr-1*) as a negative regulator of PHA-4 binding within the pharynx, but *emr-1* did not modulate PHA-4 binding in the intestine. Upon promoter association, PHA-4 induced large-scale chromatin de-compaction, which, we hypothesize, may facilitate promoter access and productive transcription. Our results reveal two tiers of PHA-4 regulation. PHA-4 binding is prohibited in intestinal cells, preventing target gene expression in that organ. PHA-4 binding within the pharynx is limited by the nuclear lamina component EMR-1/emerin. The data suggest that association of PHA-4 with its targets is a regulated step that contributes to promoter selectivity during organ formation. We speculate that global re-organization of chromatin architecture upon PHA-4 binding promotes competence of pharyngeal gene transcription and, by extension, foregut development.

## Author Summary

Central regulators of cell fate establish the identity of cells by direct regulation of large cohorts of genes. In *Caenorhabditis elegans*, foregut (or pharynx) identity relies on the FoxA transcription factor PHA-4, which activates different target genes in different cellular environments. An outstanding question is how PHA-4 distinguishes between target genes for appropriate transcriptional control. Here we examine PHA-4 interactions with target promoters in living embryos and with single-cell resolution. While PHA-4 was found throughout the digestive tract, binding and activation of pharyngeally expressed promoters was restricted to a subset of pharyngeal cells and excluded from the intestine. An RNAi screen identified emerin (*emr-1*) as a negative regulator of PHA-4 binding within the pharynx. Upon promoter association, PHA-4 induced large-scale chromatin de-compaction, which, we hypothesize, facilitates promoter access. Our results reveal two tiers of PHA-4 regulation. PHA-4 binding is prohibited in intestinal cells and is limited in the pharynx by the nuclear lamina component EMR-1/emerin. The data suggest that association of PHA-4 with its targets is a regulated step that contributes to promoter selectivity during organ formation. We speculate that global re-organization of chromatin architecture upon PHA-4 binding promotes competence of pharyngeal gene transcription and, by extension, foregut development.

## Introduction

Selector genes govern the fates of groups of cells related to each other by virtue of their cell type, position or affiliation to an organ [1]. Genomic methods have revealed that selector genes directly control hundreds, even thousands, of target genes, which define the characteristics of a particular cell type [2]–[6]. For example, the mesodermal factor Twist regulates genes that control mesodermal behaviors including gastrulation, migration and proliferation [7]. The myogenic regulatory factor MyoD directly activates skeletal muscle genes during both early cell-fate specification and later differentiation [4], [8]. The global regulatory strategy of selector genes raises the question of how targets of broadly active selector genes are expressed selectively at the appropriate times and places.

The selector gene *pha-4/FoxA* plays a broad role in the development and physiology of the *C. elegans* digestive tract. PHA-4 establishes the diverse cell types of the *C. elegans* pharynx during early embryogenesis, and drives differentiation and morphogenesis at later stages [9]–[12]. After birth, PHA-4 is required for growth and gonadogenesis in larvae [2], [13]–[15] and promotes longevity in adults [16], [17]. The targets of PHA-4 are likely distinct in different tissues and at different developmental stages. For example, numerous PHA-4 target genes have been identified within the pharynx, but most of these are not active in the intestine or gonad [2], [11], [18]. Recent chromatin immunoprecipitation data with tagged PHA-4 suggest different genes are bound by PHA-4 at different developmental stages [19]. How is appropriate regulation of PHA-4 target genes achieved? One mechanism is combinatorial control by PHA-4 with other transcription factors. A single PHA-4 binding site is not sufficient for transcriptional activation, and most foregut promoters carry four or more cis-regulatory elements that contribute towards appropriate spatial and temporal expression [13], [18], [20]–[25]. In addition, DNA binding affinity of PHA-4 for target genes modulates the timing of activation [2], [18]. High affinity sites promote earlier transcriptional onset compared to lower affinity sites, within the context of the intact cis regulatory region [2]. These studies suggest that binding affinity, feed-forward loops, positive feedback and combinatorial control, are necessary to achieve accurate temporal gene expression. However, it is still largely unknown how spatial regulation is accomplished. For example, why are pharyngeal genes active in the pharynx but not in the intestine, despite the widespread expression of PHA-4 in both organs?

Studies have implicated the nuclear periphery for modulation of gene transcription. Active and inducible genes are recruited to nuclear pores [26]–[30]. Conversely, nuclear lamins and their associated proteins have been associated with transcriptional repression and chromatin organization [31]–[36]. Inactive genes are often positioned at the nuclear lamina [37], and tethering of genes to the nuclear lamina can reduce expression levels [38], [39]. This effect is not comprehensive, however, as some peripherally-located genes are active [38]–[41]. These results indicate that the nuclear lamina is transcriptionally competent, and raise the question of the nature and degree of lamina-mediated repression.

The nuclear lamina of *C. elegans* is composed of a single B-family lamin (*lmn-1*; [34], [42], three associated LEM proteins [43] and additional factors [44]. Loss of LMN-1 leads to embryonic arrest by the 300-cell stage, with chromosome bridges between sister cells [34]. Inactivation of the LEM protein *emr-1/Ce-emerin* has no obvious phenotype on its own and produces viable animals, but inactivation of both *emr-1* and a second LEM protein *man-1*/Ce-MAN1, causes lethality at around the 100 cell stage with phenotypes similar to those of *lmn-1* [43], [45]. Barrier to autointegration factor BAF-1 is a fourth lamina protein required for chromosome segregation and integrity of the lamina [46], [47]. BAF-1 associates with *cis-*regulatory sites within the promoters of *eff-1* and *aff-1,* and is required to repress *eff-1* expression in epidermal seam cells [31]. These data implicate the *C. elegans* nuclear lamina for transcriptional repression, but the mechanism is unknown.

In this study, we probe the role of PHA-4 for pharyngeal gene activation, using artificial chromosomes to monitor PHA-4 binding and activity in living embryos [48]–[52]. We find that PHA-4 associates with its targets long before their activation. This association is restricted to a subset of pharyngeal cells, despite the ubiquitous expression of PHA-4 throughout the digestive tract, and is modulated by the nuclear lamina protein EMR-1/Emerin. Binding of PHA-4 leads to extensive chromatin decompaction and repositioning, in a process that precedes transcription. Previous studies implicated mammalian FoxA factors for local opening of chromatin and inhibition of linker histones [53]. Our data suggest that, in addition to local alterations, FoxA factors can induce large-scale changes in chromatin architecture, which may contribute to the long-range effects of FoxA proteins on transcription and recombination [54], [55]. These studies provide a framework for understanding the cell-type biases of selector genes for their targets.

## Results

### *pax-1* is expressed in a subset of pharyngeal cells, and its expression is regulated by cis-regulatory elements that cooperate with PHA-4/FoxA

Our goal was to explore PHA-4 association with its target genes in living embryos. We chose to analyze *myo-2*, which is a well-characterized gene expressed exclusively in pharyngeal muscles [56], [57], and *pax-1*, which we show below is a PHA-4 target expressed in the pharyngeal marginal cells and some other pharyngeal cell types. To initiate the study, we characterized *pax-1* cis-regulatory sites for pharyngeal expression.

**Figure 1:**
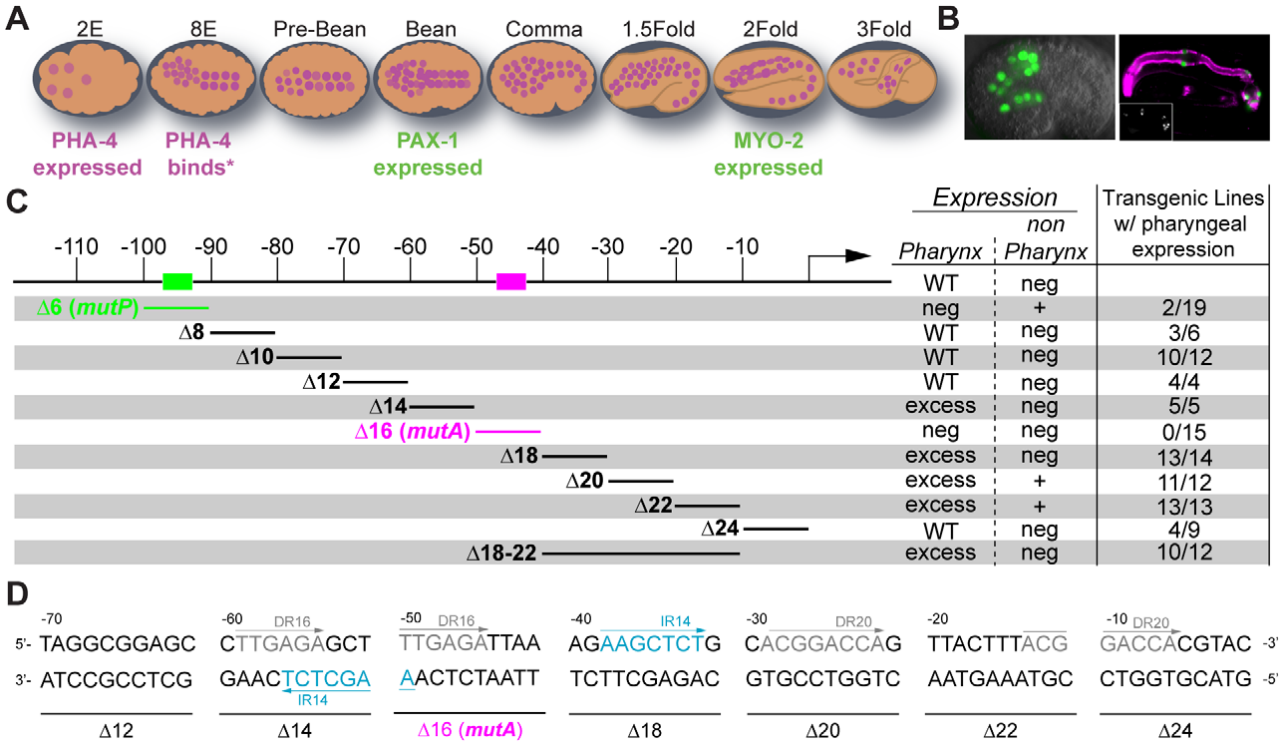
Scanning mutagenesis of the *pax-1* promoter. (A) A cartoon depicting the pattern of PHA-4 expression during different stages of embryogenesis. Embryonic events that occur at specific developmental stages are annotated. PHA-4 expression from [9] [10], *myo-2* expression from [99] and *pax-1* expression from this study. (B) *pax-1*::GFP expression in 14 pharyngeal cells in a comma stage embryo (left) and GFP (green; anti-GFP Molecular Probes) co-stained with anti-intermediate filament antibody (magenta, right, [95]). Marginal cells and pm8 are visible, but e2 cells are faint in this image. GFP alone shown in the inset. (C) Linker scanning mutagenesis reveals two positive cis-regulatory sites: *mutP* (green) and *mutA* (magenta).% pharyngeal expression: number of independent lines with pharyngeal expression/total number of independent lines analyzed. Neg: negative. WT: wild type. (D) The architecture of the *pax-1* promoter 70 bp upstream to the TSS revealing direct repeats (DR in grey) and inverted repeats (IR in blue).

To analyze *pax-1*, we constructed two GFP reporters: a translational fusion within the second exon of *pax-1* (PAX-1::GFP; Table S1A) and a transcriptional fusion between GFP and the *pax-1* translation initiation site (*pax-1*::GFP). These constructs revealed that *pax-1* was expressed in 14 pharyngeal cells, which included nine marginal cells, the e2 epithelial cells and the pm8 muscle, based on morphology, position and co-staining for marginal cell filaments (Figure 1B, Figure S1). We focus on the marginal cells here. Expression of *pax-1*::GFP in marginal cells was first detectable in two rows of pharyngeal nuclei shortly after embryonic cell division ceased, at the late-bean to early-comma stages of development (). Expression gradually faded during later embryogenesis and was undetectable in larvae or adult worms.

Examination of the *pax-1* promoter revealed a consensus PHA-4 binding site between −92 and −98 base pairs (bp) upstream of the transcriptional start site (Figure 1C). Gaudet *et al.* previously showed that three copies of this site were sufficient to activate expression of a heterologous promoter within pharyngeal cells [18]. Conversely, we found that deletion () or mutation (*MutP* Figure 1C) of the predicted PHA-4-binding site eliminated *pax-1*::GFP expression in 17/19 transgenic lines (Figure 1C). We speculate that the 2/19 lines with residual pax-1::GFP expression in pharyngeal cells may be activated by cryptic enhancers originating from nearby sequences in the array. Interestingly, in addition to loss of pharyngeal expression, the mutant reporters exhibited significant ectopic GFP in the epidermis (Figure 1C and Figure S3). Together, these results suggest the PHA-4 binding site is required to activate expression in the pharynx and repress expression in epidermal cells.

To identify additional cis-regulatory sites within the *pax-1* promoter, we performed linker-scanning mutational analysis beginning −115 bp upstream of the *pax-1* transcriptional start site (Figure 1C). This survey revealed a second activation site within Delta16, which we will refer to as *mutA*: replacement of 10 bp from *mutA* abolished all GFP reporter expression (TTGAGATTAA; Figure 1D). Scanning mutagenesis also uncovered two negative regulatory regions. First, mutations in either Delta14 or Delta18 generated a high proportion of transgenic lines that expressed *pax-1::GFP* in additional pharyngeal cells, to approximately 20 cells (Figure S4A). Second, mutations in Delta20, and to a lesser degree Delta22, lead to GFP^+^ cells outside of the pharynx (Figure S4B). In sum, mutational analysis revealed both positive and negative cis-regulatory sites that cooperated with PHA-4 to activate *pax-1* within pharyngeal marginal cells. A direct repeat (TTGAGA) lies within Delta14 and Delta16, and an inverted repeat (AAGCTCT) lies within Delta14 and Delta18, suggesting one or both of these may be recognition sites for transcription factors (Figure 1D). These cis-regulatory sites provided a means to examine the role of PHA-4 for pharyngeal gene activation, described below.

### PHA-4 binds to its pharyngeal targets hours before the onset of gene expression

The mutational analysis suggested that both *myo-2* and *pax-1* were direct PHA-4 target genes. To test this idea further, we used the Nuclear Spot Assay (NSA) to examine association of PHA-4 with pharyngeal promoters *in vivo*. The NSA allowed us to track PHA-4 binding to promoters in living embryos, with precise spatial and temporal resolution. For this assay, we constructed a transgene array or “pseudo-chromosome” that carried multiple copies of a target promoter and the Lac operator [48]–[52], [58]. A co-selectable marker (to identify transgenic animals) and herring sperm genomic DNA (to provide sequence complexity without added *C. elegans*' sequences [59]) were also included. The pseudo-chromosome arrays carried fusions of CFP::LacI and PHA-4::YFP; CFP::LacI bound to LacO sequences on the arrays and revealed their position and morphology in the nucleus. PHA-4::YFP bound to its promoter appeared as a dense magenta “dot” that colocalized with CFP::LacI. Diffuse PHA-4::YFP in the background indicated binding of PHA-4::YFP to genomic loci. It was previously shown that the NSA method accurately reflected transcriptional regulation, as detected by other methods such as chromatin immunoprecipitation [50], [52]. *C. elegans* arrays are relatively stable through mitosis and meiosis, and are incorporated into chromatin [59], [60]. However, we recognize that pseudo-chromosomes are not replicas of *C. elegans* chromosomes, and they likely differ from endogenous chromosomes in some regards.

**Figure 2:**
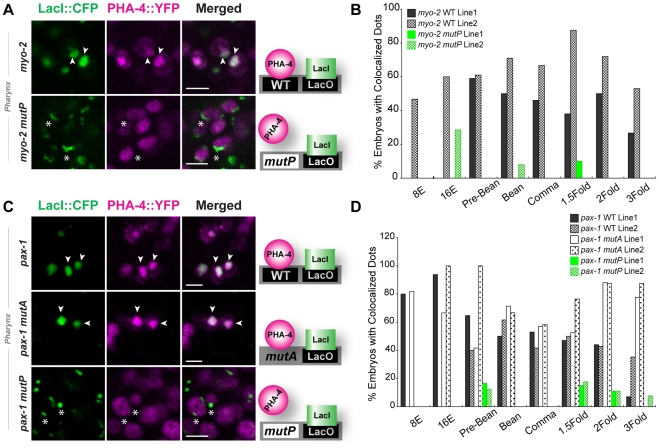
PHA-4 associates with pharyngeal target promoters by the 8E (**∼**100 cell) stage. CFP::LacI (depicted in green) and PHA-4::YFP (depicted in magenta) co-localization on pseudo-chromosomes bearing (A) *myo-2* or (C) *pax-1* promoters. Merge is white. Binding to pseudo-chromosomes is abolished by mutating the PHA-4 binding sites in *myo-2 mutP* or *pax-1 mutP* but is not affected when an unrelated activation site is mutated (*pax-1 mutA*). The cartoon illustrates the interpretation of the data. (B, D) Quantitation of embryos with co-localized CFP::LacI and PHA-4::YFP in transgenic lines bearing (B) a wild-type *myo-2* promoter (solid and hatched black, WT) or one with mutated PHA-4 sites (solid and hatched green, *mutP*) and (D) a wild-type *pax-1* promoter, a mutant promoter lacking PHA-4 binding sites (*pax-1 mutP*) or a mutant promoter inactivated for an unrelated activation site (*pax-1 mutA)* (white and dotted, *mutA1*). Numbers of embryos scored per stage shown in Figure S5C. Scale bar, 3 microns. Arrowheads indicate PHA-4 bound (co-localized) pseudo-chromosomes. Asterisks indicate arrays that lack associated PHA-4::YFP.

We observed multiple pharyngeal cells with PHA-4::YFP enriched on pseudo-chromosome arrays, supporting the notion that *myo-2* and *pax-1* are direct PHA-4 targets (Figure 2 and an additional third target *C44H4.1*: Figure S5A). The association was detected by the 8E (endodermal) stage, which was the earliest stage we could visualize PHA-4::YFP (∼100 cells). The proportion of embryos with associated PHA-4::YFP remained relatively constant until the two-fold (*pax-1*) or three-fold (*myo-2*) stages. The robust association of PHA-4 to its target promoters required a consensus PHA-4 binding site since pseudo-chromosome arrays that carried a promoter with mutated PHA-4 binding sites [2] failed to recruit PHA-4::YFP (Figure 2). These data reveal that PHA-4 bound target promoters long before they were transcriptionally active, indicating that PHA-4 occupancy did not correlate with transcriptional activity per se, but rather with transcriptional potential. Similarly, vertebrate FoxA2, which is orthologous to PHA-4, binds the albumin promoter in mouse endodermal cells long before the gene is active [61].

### PHA-4 binding leads to large-scale chromatin decompaction that is largely independent of transcription

What are the repercussions of PHA-4 association to target genes? Previous genetic studies suggested that PHA-4 and its orthologues influence the chromatin environment [11], [53], [62]–[64]. For example, PHA-4 recruits the histone variant HTA.Z/HTZ-1 to a subset of pharyngeal promoters, including that of *myo-2* [63], and it interacts genetically with predicted chromatin regulators [11], [63]. Vertebrate orthologues of PHA-4 associate with chromatin and can block compaction by H1 histones [53], [54], [65]. These observations prompted us to examine the chromatin morphology of the pseudo-chromosome arrays.

**Figure 3:**
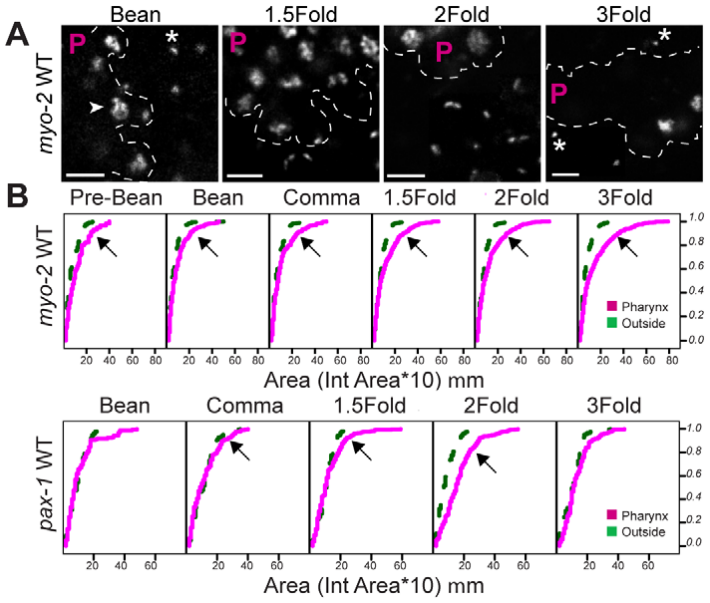
Decompaction of pseudo-chromosomes during pharyngeal differentiation. (A) Pseudo-chromosomes bearing wild-type *myo-2* promoters within the pharynx (P region) or outside, at the indicated stages. Decompacted (arrow) and compacted (asterisk) pseudo-chromosomes are noted. PHA-4::YFP was used to identify pharyngeal cells (not shown). Scale bar, 3 microns. (B) Cumulative distributions of areas for pseudo-chromosomes bearing wild-type *myo-2* or *pax-1* promoters at the indicated developmental stages. The horizontal axis represents the area of individual pseudo-chromosomes multiplied by 10. The vertical axis represents the cumulative proportion of pseudo-chromosomes with an equal or smaller area. Curves shifted to the right, indicate a greater proportion of pseudo-chromosomes with large areas, for pharyngeal cells (magenta) relative to cells outside of the pharynx (green). Areas of pseudo-chromosomes increased as embryos developed (p = 0.00003 for *myo-2*, p = 0.0002 for *pax-1)*. For *myo-2*, n = 2 lines, 10 embryos per stage per line. For *pax-1*, n = 1 line, 5 embryos per stage.

**Figure 4:**
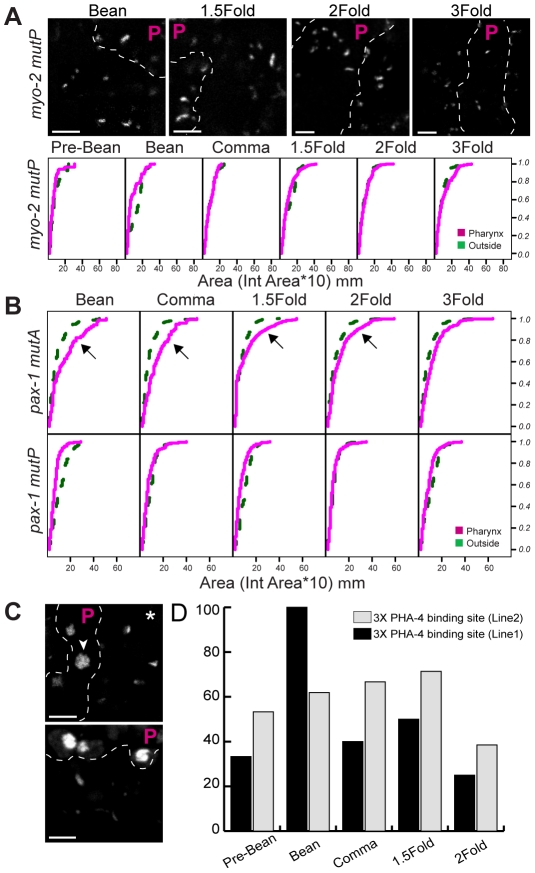
PHA-4 is required for chromatin decompaction. (A) Pseudo-chromosomes bearing mutated PHA-4 binding sites within *myo*-2 either within the pharynx (P region) or outside, at the indicated stages. PHA-4::YFP was used to identify pharyngeal cells (not shown). Scale bar, 3 microns. Cumulative distributions of pseudo-chromosome areas for (A) mutant *myo-2* or (B) mutant *pax-1* pseudo-chromosomes. Lines analyzed were mutated for PHA-4 binding sites within *myo*-2 (*MutP*), the PHA-4 binding site within *pax-1* (*MutP*) or an alternative activation site within *pax-1* (*mutA*). The horizontal axis represents the area of individual pseudo-chromosomes multiplied by 10. The vertical axis represents the cumulative proportion of pseudo-chromosomes with an equal or smaller area. Note the overlap of pseudo-chromosome areas for PHA-4-binding mutations within the pharynx (magenta) and outside of the pharynx (green), indicating no induced decompaction. For *myo-2*, n = 2 lines, 10 embryos per stage, per line. For *pax-1*, n = 1 line each mutant, 5 embryos per stage. (C) Pseudo-chromosomes bearing 3X PHA-4 binding site repeats within the pharynx (P region) or outside, at the bean stage (upper) and 2-Fold stage (lower). PHA-4::YFP was used to identify pharyngeal cells (not shown). Scale bar, 3 mm. Note the decompaction with pharyngeal cells (arrowheads) relative to non-pharyngeal cells (asterisk). (D) Quantitation of embryos with co-localized CFP::LacI and PHA-4::YFP in transgenic lines bearing 3X repeats.

We observed progressive decompaction of pseudo-chromosomes bearing wild-type *myo-2* or *pax-1* promoters, detectable as a large, diffuse dot (Figure 3A). We quantified the changes by measuring the areas of individual pseudo-chromosomes and analyzing the areas with Cox regression models (Materials and Methods). This analysis revealed that both the number of decompacted pseudo-chromosomes and the degree of decompaction increased over time (Figure 3B, Table S2, Table S3). This effect was observed within pharyngeal cells, the eventual site of *myo-2* and *pax-1* expression, but not in non-pharyngeal cells (Figure 3). Cumulative areas were larger in the pharynx compared to “outside” the pharynx as early as the pre-bean stage for *myo-2* (p = 7×10^−11^) and the bean stage for *pax-1* (p = 0.007). For *myo-2*, many pseudo-chromosomes became decompacted prior to transcription at the 2-fold stage and remained decompacted. For *pax-1*, decompaction began at the comma stage and was maximal at the 1.5 and 2-fold stages, when *pax-1* is transcribed (Figure 3B). In sum, *pax-1* and *myo-2* pseudo-chromosomes underwent decompaction preceding and during transcription within the pharynx. In contrast, pseudo-chromosomes in which PHA-4 binding sites had been mutated behaved similarly in pharyngeal and non-pharyngeal cells, with little increase in size over time (Figure 4). These observations indicate that PHA-4 is required for large-scale decompaction of chromatin in extragenic arrays.

We considered three spurious reasons for changing pseudo-chromosome areas, independent of PHA-4. First, we examined whether array sizes were a consequence of expanding nuclear size. However, nuclear size remained relatively constant at the stages assayed in this study, and no normalization to nuclear size was necessary (Figure S5D). Second, we tested whether decompaction reflected an artificial interaction between LacI and PHA-4. However, PHA-4 binding and consequent decompaction of pseudo-chromosomes was observed in transgenic lines lacking LacI protein (Figure S5B). Third, we wondered if 3D volumetric measurements would be more accurate than the 2D area measurements used here. 3D analysis was subject to photobleaching of the YFP signal while collecting Z-stacks, which hindered 3D reconstruction. A comparison of area versus volume measurements in embryos with minimal photobleaching revealed a similar trend in array expansion (Figure S7). These controls suggest that array decompaction reflects PHA-4 interactions with target chromatin.

PHA-4 is a critical regulator of pharyngeal gene transcription, and transcription is often associated with chromatin decondensation [66]–[69]. We therefore tested whether decompaction of pseudo-chromosome arrays in pharyngeal cells reflected PHA-4 binding or transcriptional activity. When the PHA-4 binding site is mutated in the *myo-2* promoter, *myo-2* is still transcribed, but at a later developmental time [2]. We observed little pseudo-chromosome decompaction for arrays bearing this mutant promoter (Figure 4). Residual decompaction was observed at the 3-fold stage, which may reflect transcriptional activity. This result suggests that PHA-4 is a critical contributor to large-scale decompaction of *myo-2,* especially at early developmental stages. Conversely, we examined pseudo-chromosome arrays bearing mutant *pax-1 mutA* promoters, which were no longer transcribed but which bound PHA-4. These arrays became decompacted despite the absence of productive transcription (inside versus outside the pharynx (p<0.0001)), whereas *pax-1 mutP* arrays bearing a mutated PHA-4 binding site did not (Figure 4B) (smaller inside vs. outside the pharynx p = 2×10^−14^). This result suggests that productive transcription is not essential for decompaction, and that PHA-4 association is sufficient. To test this idea more stringently, we created arrays bearing three repeats (3X) of a PHA-4 binding site derived from *pax-1*, but lacking additional promoter sequences. The 3X repeats were sufficient for PHA-4::YFP recruitment to the pseudo-chromosome and caused large-scale decompaction (Figure 4C). These data reveal that PHA-4 binding, more than ongoing transcription, induces large-scale reorganization of chromatin in developing *C. elegans* embryos.

### PHA-4 binding is spatially regulated

**Figure 5:**
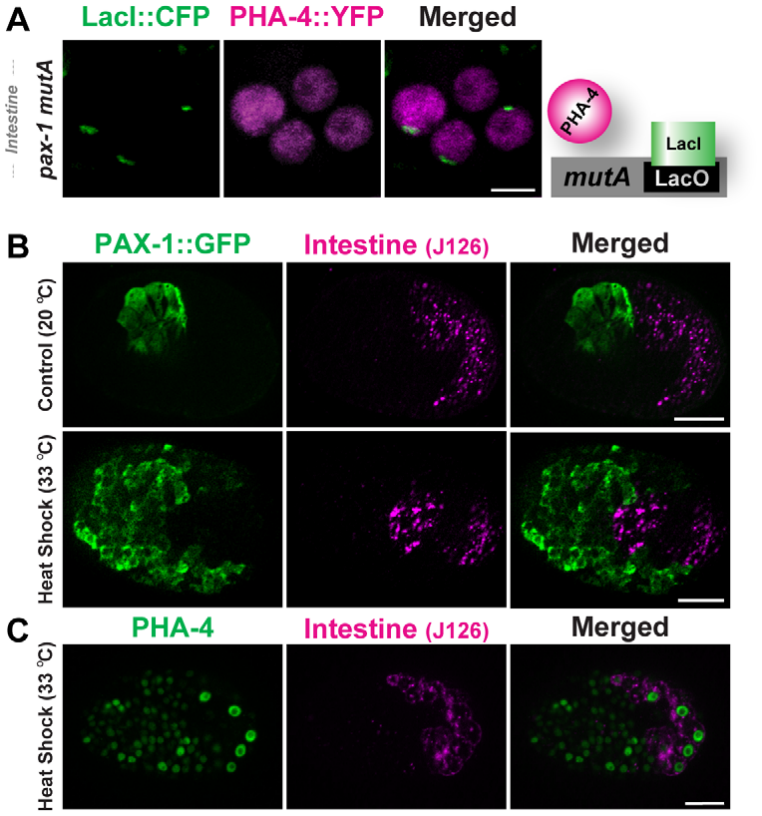
PHA-4 binding and activity is limited in the intestine. (A) PHA-4::YFP (depicted in magenta) does not associate with pseudo-chromosomes (marked with CFP::LacI, green) bearing the *pax-1 mutA promoter* in the intestine. The cartoon illustrates the interpretation of the data. (B) Over-expression of PHA-4 under a heat-shock promoter leads to widespread expression of PAX-1::GFP (green) in multiple tissues but not in the intestine (magenta; J126 (lower panel). Control embryos that did not receive heat-shock express PAX-1::GFP only in marginal cells (upper panel). (C) PHA-4 is expressed in all tissues after heat shock, including the intestine (magenta; J126). Scale bar, 10 microns.

PHA-4 is expressed broadly, including the pharynx, intestine, rectum, somatic gonad and some neurons [9], [10], [14], [16], [70], yet PHA-4 targets are activated in discrete cell-types. For example, *pax-1* is expressed in marginal cells but not in the intestine (Figure 1). We wondered if the discriminate activation of downstream targets could be explained by regulated binding of PHA-4. PHA-4 binding was surveyed in a transgenic line carrying the *pax-1 mutA* promoter at three developmental stages (bean, comma and 1.5-fold) in one mid-section focal plane. Pharyngeal binding was detected in ∼67% of embryos at the bean stage (10/15), ∼58% at the comma stage (7/12), and ∼76% at the 1.5-fold stage (13/17; average 68%). By contrast, binding was almost never detected in the intestine at any stage (<1%; 0/44 embryos counted; additional embryos surveyed but not counted; Figure 5A). Similar results were observed with arrays bearing *myo-2* (data not shown). An optical section through a 1.5-fold embryo sampled approximately 10 pharyngeal nuclei and 10 intestinal nuclei, indicating that the differential association of PHA-4 did not reflect different numbers of nuclei in each organ.

Does regulated binding lead to differential PHA-4 activity in disparate tissues? To answer this question we induced ectopic PHA-4 using a heat-shock promoter in transgenic lines bearing *pax-1::GFP*. HS::PHA-4 induced widespread expression of *pax-1*::GFP in many cells. However, we did not observe *pax-1*::GFP in the developing intestine (0/50) (Figure 5B). This absence did not reflect variable PHA-4 expression, since antibody staining demonstrated that PHA-4 was expressed in intestinal cells equivalently to other tissues after heat shock (Figure 5C). We detected no ectopic expression of the GFP reporter in non-heat shocked embryos (Figure 5B), nor did we observe ectopic expression when we tested *HS::pha-4DeltaDBD* [9], which lacked the DNA binding domain (data not shown). These findings indicate that PHA-4 binding to pseudo-chromosome arrays limits PHA-4 activity, and that both binding and activity are sensitive to the cellular environment. This conclusion agrees with previous observations that HS::PHA-4 can induce embryonic cells to convert to a pharyngeal fate, but that the intestine is immune to ectopic PHA-4 [9].

### The integral nuclear membrane protein *emr-1* regulates PHA-4 binding to targets in the pharynx

To begin to understand the selective binding of PHA-4 in different cell types, we conducted a small RNAi screen for nuclear factors that modulate PHA-4 binding to target promoters. We used SM1634 carrying a mutant *pax-1* promoter because *pax-1*-containing arrays typically bound PHA-4::YFP in fewer pharyngeal cells than *myo-2*-containing arrays (data not shown). We surveyed genes involved in chromatin modification such as histone demethylation, methylation, acetylation and RNA interference. Given the proximity of the pseudo-chromosomes to the nuclear lamina, we also tested genes involved in nuclear envelope structure and function. We counted the number of nuclei with bound PHA-4::YFP in a section that passed through the pharynx and intestine of comma to 1.5 fold embryos.

**Figure 6:**
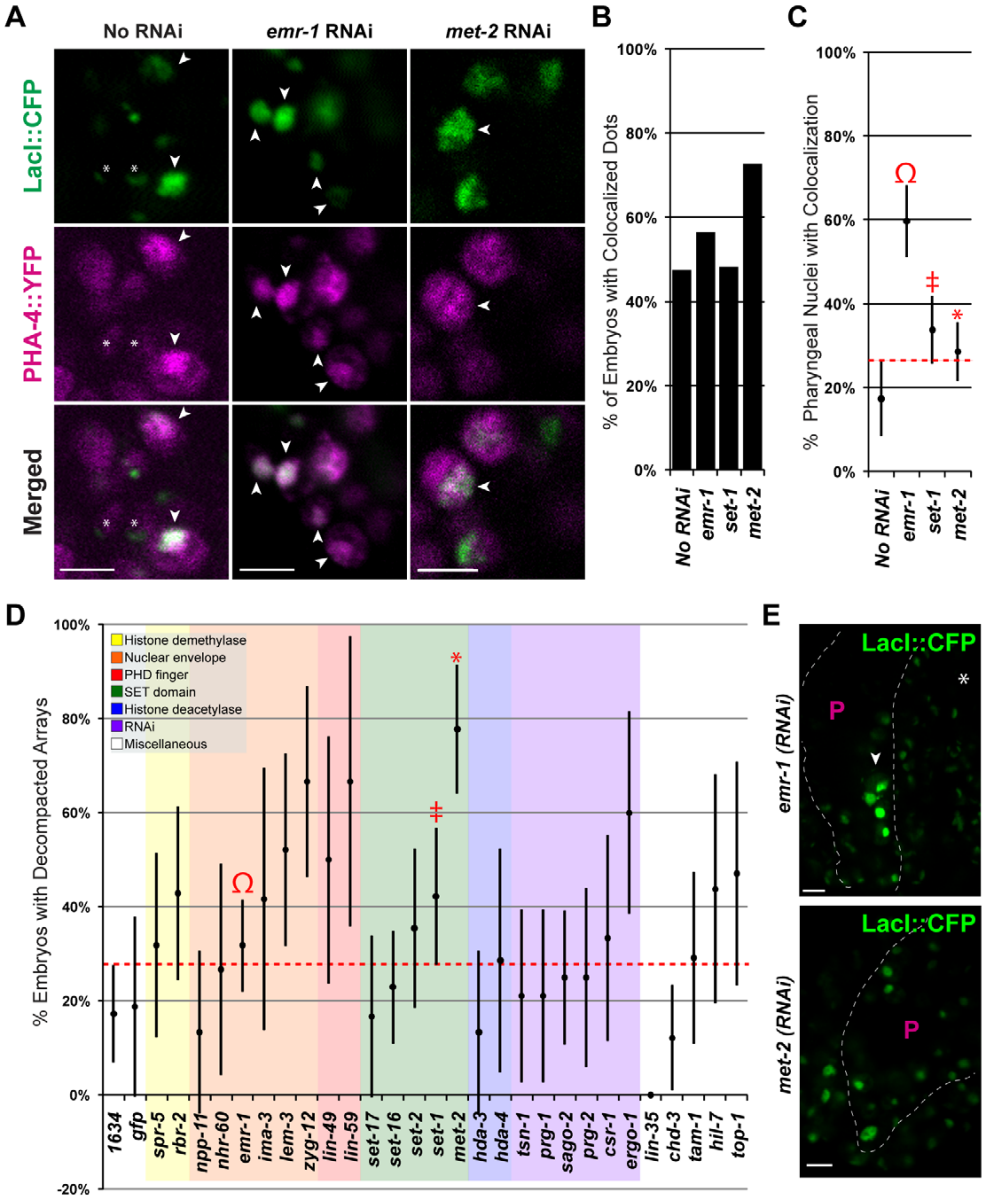
Emerin inhibits PHA-4 binding in the pharynx. (A) CFP::LacI (depicted in green) and PHA-4::YFP (depicted in magenta) co-localization on pseudo-chromosomes bearing *pax-1MutA* after No RNAi, *emr-1(RNAi)* or *met-2(RNAi)*. (B) Percentage of embryos with at least one co-localized dot (No RNAi 9/19, *emr-1(RNAi)* 13/23, *set-1(RNAi)* 14/29, and *met-2(RNAi)* 16/22) (C) Percentage of pharyngeal nuclei with bound PHA-4::YFP among embryos with co-localization. After *emr-1* reduction (Ω, n = 13), embryos had a ∼3 fold increase in PHA-4::YFP binding compared to No RNAi controls (n = 9). For *met-2* and *set-1* n = 16 and 14, respectively D) The proportion of embryos bearing de-condensed arrays for each RNAi treatment is graphed. See Table S2 for the number of embryos assayed. (E). Example of pseudo-chromosomes after *emr-1(RNAi)* or *met-2(RNAi)*. Decompaction within the pharynx (arrowhead) presumably reflects PHA-4 association. Scale bar, 3 microns.

Of 28 genes surveyed, *emr-1*/Emerin had the most dramatic effect on PHA-4 binding (Figure 6). In the control, almost half of embryos had at least one nucleus with PHA-4::YFP bound to the *pax-1* promoter, with an average of 17% pharyngeal nuclei bound within an embryo (Figure 6B and 6C). Inactivation of emerin lead to a large increase in the number of pharyngeal nuclei with bound PHA-4::YFP, to ∼60% (Figure 6A and 6B). Although EMR-1 is widely expressed in all embryonic tissues [36], we observed binding only in the pharynx and not in the intestine of *emr-1(RNAi*) embryos (1 of 88 embryos (1.25%) in three experiments). These results reveal that the nuclear lamina interferes with binding of PHA-4::YFP to its targets within pharyngeal cells, but that additional processes function in the intestine.

*emr-1(RNAi)* strongly lowered expression of EMR-1 protein (Figure S6), and promoted pseudo-chromosome decompaction compared to wild-type embryos, raising the possibility that increased binding of PHA-4 in *emr-1(RNAi)* embryos could be a consequence of increased accessibility. 32% (28/88) of *emr-1(RNAi)* embryos had decondensed arrays compared to 17% (9/52) for wild-type (Figure 6A and 6D). To explore the role of decompaction, we examined other genes for effects on pseudo-chromosome morphology and PHA-4::YFP binding. RNAi against 6 additional genes caused a global de-condensation of pseudo-chromosomal arrays at the comma to 1.5-fold stages of embryogenesis (*lem-3, zyg-12, lin-59, set-1, met-2, ergo-1* Figure 6A and 6D). The arrays in these embryos appeared more distended and were brighter than wild-type embryos suggesting increased CFP::LacI expression. The de-condensation of the pseudo-chromosomes was not restricted to a specific tissue, but was observed in most nuclei in an optical section across the embryo. RNAi against *set-1*, a gene encoding a potential SET-domain methyltransferase [71], caused global decompaction in 19 of 45 embryos (40%, Figure 6D). The decompaction observed in *set-1*(*RNAi*) embryos was not surprising given that *set-1* has been implicated in transgene silencing [72]. However, *set-1(RNAi)* embryos did not lead to increased PHA-4::YFP association (Figure 6B and 6C). The most dramatic effect was observed for *met-2*, a histone H3 lysine 9 dimethyltransferase that is homologous to human SETDB1 [73] [74]. Arrays appeared more decompacted in *met-2(RNAi)* nuclei, and a greater proportion of arrays were decondensed compared to those in wild-type embryos (77%, 28/36, Figure 6A, 6D, and 6E). Decompaction by reduced *met-2* had some effect on PHA-4 binding since a greater proportion of *met-2(RNAi)* embryos had PHA-4::YFP localized to pseudo-chromosomes (Figure 6C). However, within those embryos, only 29% of pharyngeal nuclei had PHA-4::YFP bound to pseudo-chromosomes, and this difference was not statistically significant from control. (Figure 6A and 6C). These data indicate that general decompaction may influence PHA-4::YFP association, but that Emerin likely modulates PHA-4::YFP binding by additional mechanisms as well.

## Discussion

This study provides three insights towards understanding the regulatory strategies that drive foregut organogenesis by a selector gene. First, we have probed the association and activity of PHA-4 with its target genes in living embryos. PHA-4 binds foregut genes selectively, within the pharynx but not within a neighboring organ. This association promotes large-scale chromatin decompaction of target genes and surrounding sequences, prior to the onset of transcription. We hypothesize that opening of chromatin may facilitate productive transcription at later stages. Second, binding of PHA-4 to pseudo-chromosomes in the pharynx is restricted by the lamina-associated protein EMR-1/Emerin. The nuclear lamina induces transcriptional silencing in many organisms, but the mechanism is unclear [75]. Our study reveals that EMR-1-mediated silencing can occur by blocking transcription factor association. Third, we have defined the promoter architecture that establishes expression of the *pax-1* gene within a subset of pharyngeal cells. Expression is promoted by broadly-acting enhancer regions, which include a PHA-4 site, and limited to fourteen pharyngeal cells by repressive elements.

### PHA-4 binding leads to large-scale chromatin decompaction in living embryos

The transparency of *C. elegans* enables analysis of selector gene behavior in living embryos. Our characterization of PHA-4 and its target genes revealed regulated association with target promoters, which induced extensive chromatin decompaction in selected cells. We note that these events were visualized by single-cell analysis and would not have been detected by biochemical approaches such as chromatin immunoprecipitation. Importantly, the expression of pharyngeal reporter constructs embedded within complex DNA sequences, with few exceptions, mimics expression of the endogenous cognates, as detected by *in situ* stains [66], [76]. Thus, the bulk of gene regulatory processes are preserved in the arrays.

Large-scale decompaction of chromatin by a selector gene, which, to our knowledge, has not been observed previously, is consistent with observations regarding *pha-4/Fox* orthologues in other organisms. In breast cancer cells, global location analysis previously revealed that FoxA1 bound many regions located >50 Kb from a transcription start site [77]. FoxA1 induced both local effects, such as chromatin remodeling and transcription factor recruitment [54], but also long-range effects, such as physical interactions between enhancers and promoters [77]. In *S. cerevisiae*, Fkh1/Fox controls donor preference during mating-type switching [55]. Fkh1 promotes recombination for loci separated by 50 Kb and does so without altering transcription or local chromatin [78]. These observations suggest Fox factors in diverse organisms contribute to long-range interactions between distant loci. In our system, we estimate roughly one PHA-4 target promoter per 25 Kb of DNA within the pseudo-chromosomes. This number derives from ∼200 copies of target promoter (qPCR, data not shown) embedded in arrays of ∼5–7 Mb [79]. At endogenous loci, PHA-4::GFP associates with 4350 sites in the embryonic genome, a surprisingly high density of binding sites [19]. The distance observed in *C. elegans* is comparable to those in the yeast and mammalian studies, suggesting that large-scale, Fox-mediated chromatin re-organization might operate in all three organisms.

The association of PHA-4::YFP with pseudo-chromosomes was not constitutive, but responded to the cellular milieu in two ways. First, within the pharynx, PHA-4::YFP binding was restricted by EMR-1/emerin. EMR-1/emerin resides at the nuclear lamina, which suggests that tethering of pharyngeal genes or trans-acting factors at the nuclear periphery may modulate PHA-4 binding. We note that the nuclear lamina appears normal after *emr-1(RNAi),* and affected embryos are healthy and viable (This study and [43]). We speculate that the loss of emerin may have subtle effects on tethering or formation of heterochromatin, signaling pathways or lamina-associated proteins that alter gene activity [80]. Second, in the intestine, PHA-4 binding to *pax-1* and *myo-2* was inhibited completely, and inhibition was not relieved by *emr-1(RNAi). HS::pha-4* cannot activate *pax-1::GFP* within intestinal cells (this study) or convert nascent intestinal cells to a pharyngeal fate [9], indicating PHA-4 functions poorly in this embryonic tissue. By contrast, ubiquitous expression of the *C. elegans MyoD* homolog *hlh*-*1* induces the body-wall muscle program throughout the embryo, including developing intestinal cells [48], [81]. We suggest that the limited activity of PHA-4 within the intestine may reflect the inability of PHA-4 to associate with its pharyngeal target genes. This lack of association in the intestine may reflect the presence of gut-specific repressive systems that block pharyngeal gene activation in the intestine, or the absence of appropriate cofactors and coactivators.

What is the nature of PHA-4-induced chromatin restructuring? The global decompaction we observe is consistent with a disordered structure, such as decondensation by loss of nucleosomes and/or reconfiguring of chromatin into loops or coils [82]. Although nucleosome loss can be associated with transcription [83], our data suggest that the effect of PHA-4 is independent of productive transcription. Arrays bearing the *mutA* promoter or 3X PHA-4 binding site repeats recruit PHA-4::YFP and undergo decompaction, in the absence of GFP production. In *Drosophila,* nucleosomes are lost rapidly at heat-shock loci prior to transcription, and this loss extends across several kilobases upstream and downstream of the activated gene [84]. Transcription-independent decondensation of chromatin might be required to “clear the way” for RNA Pol II, enabling cells to activate gene expression rapidly and respond promptly to developmental and environmental cues.

### Cis-regulatory architecture of the *pax-1* gene

An interesting feature of *pax-1* expressing cells is that they share a lineage relationship. We identified 11 of the 14 pax-1::GFP^+^ cells unambiguously, and found that each of these cells derived from the posterior daughter of the penultimate cell division (“px” cells; Figure S1). For example, ABaraaapapa generates a marginal cell that expresses *pax-1*. Previous studies have shown that *C. elegans* embryos are patterned according to antero-posterior (A-P) cell divisions in which pairs of A-P siblings are distinguished by high (anterior) or low (posterior) levels of nuclear POP-1, a TCF transcription factor ([85], [86] reviewed in [66]). Loss of POP-1 asymmetry alters cell fate decisions, suggesting transcriptional regulation by POP-1 confers anterior or posterior identity after each cell division [85], [86]. However, few transcriptional targets of POP-1 are known. We considered an appealing model that POP-1 might regulate *pax-1* transcription directly during the penultimate cell division and thereby contribute to A–P fate distinctions. However, none of the cis-regulatory sites we identified are a good fit with the canonical TCF binding site G(A/T)(A/T)CAAAG [87]. Thus, the relationship between *pax-1* and A-P specification remains a mystery.

Our promoter analysis identified four regulatory elements that establish *pax-1* expression in fourteen pharyngeal cells. The first was an enhancer element likely recognized by PHA-4 and defined by D6. PHA-4 can bind this sequence *in vitro* [2] and *in vivo* (this study). Moreover, this site is required for pharyngeal expression (this study), and multimers of this sequence respond to PHA-4 *in vivo* [18]. This result supports the notion that many genes expressed within the pharynx are direct targets of PHA-4 [2]. Surprisingly, while mutation of the predicted PHA-4 binding site eliminated *pax-1* expression within the pharynx, it also led to ectopic expression in non-pharyngeal cells such as epidermis. *M05B5.2* and *T05E11.3* are two additional PHA-4 target genes [2], and these also exhibited epidermal expression when the PHA-4 site was mutated to random sequences (J. Gaudet, pers. comm.). A likely possibility is that this site functions as a repression element in non-pharyngeal epithelia. PHA-4 is not expressed in the epidermis, leaving open the identity of the factor that represses epidermal expression. RNAi of the other *C. elegans* Fox genes did not result in ectopic expression in lines carrying the wildtype M05B5.2 reporter (J. Gaudet, unpublished). This result suggests that multiple Fox proteins function redundantly to repress epidermal expression, or alternatively, that an unrelated protein acts through the predicted PHA-4 binding site.

A second enhancer element defined by Delta16 contributes to *pax-1* activation. The Delta16 region contains a match to a GATA-2,3 binding site (AGATTA; [88], [89]. However, mutation of AGATTA to CTGCAG does not inactivate *pax-1* expression, suggesting this site is not recognized by a GATA factor (J.S., data not shown). We note that the sequence TTGAGA lost in Delta16 is half of a direct repeat, with a second copy located within Delta14 (Figure 1). Abutting Delta16 sequences, mutations Delta14 and Delta18 each lead to *pax-1* expression in extra pharyngeal cells. These sequences carry an inverted repeat AGAGCT that is lost in Delta14 or Delta18 (Figure 1). Two additional elements, defined by Delta20 and Delta22/Delta24, functioned negatively to restrict *pax-1* expression. A direct repeat (ACGGACCA) lies within these sequences, with one copy entirely within Delta20 and a second spanning Delta22 and Delta24. An appealing model is that PHA-4 promotes expression within the pharynx in combination with Delta16 sequences. The broad activation is refined by the repression elements embedded in Delta14/Delta18 and Delta20/Delta22/Delta24. The combination of four cis-regulatory sites explains why pan-pharyngeal PHA-4::YFP can bind its target promoters, yet those targets become transcriptionally active in only a subset of pharyngeal cells and after PHA-4 is first expressed.

We have demonstrated that the master regulator PHA-4 binds to its pharyngeal targets hours before the onset of gene expression. PHA-4 binding and activity is restricted in the intestine and negatively regulated by EMR-1/emerin in the pharynx. The association of PHA-4 with target promoters led to large-scale chromatin decompaction, which may facilitate chromatin-associated processes such as transcription. These *in vivo* results expand our understanding of PHA-4/FoxA function in driving pharyngeal transcriptional programs. Moving beyond the Nuclear Spot Assay, it will be interesting to investigate the binding and down-stream consequences of PHA-4 in its native environment, at endogenous loci.

## Materials and Methods

### Strains and growth conditions

Strains were maintained as described in [90], at 20°C, and were provided by *Caenorhabditis* Genetics Center, which is funded by the NIH National Center for Research Resources (NCRR), unless stated otherwise. Bristol N2 was used as the wild-type strain. The following mutation was used LGIV: *cha-1(p1182)*. For *pax-1::GFP* analysis the following transgenic strains were used: SM202 *pxls2(pax-1::GFP + pRF4)*, SM699 *N2(pax-1::GFP + pRF4)*, SM707 *N2(pax-1 mutP-pro::GFP + pRF4)*, SM658 *N2(pax-1 mutA-pro::GFP + pRF4),* SM660 *N2(pax-1 Delta14-pro::GFP),* SM700 *N2(pax-1Delta18-pro::GFP).* For Heat Shock: SM259 *pxEx(HS::PHA-4 + pax-1::GFP + UL8::lacZ + pRF4 + 1 KB ladder + Herring Sperm DNA)* [9]. For the Nuclear Spot Assay (NSA), the following strains were used: SM1560 *cha-1(p1182); pxEx(cha-1 + his-24pro::CFP::LacI + pha-4::yfp+ lacO + Herring Sperm DNA)*, SM1476 *cha-1(p1182); pxEx(cha-1 + htz-1pro::CFP::LacI + PHA-4::YFP +myo-2proWT + lacO + Herring Sperm DNA),* SM1429 *cha-1(p1182); pxEx(cha-1 + htz-1pro::CFP::LacI + PHA-4::YFP +myo-2proWT + lacO + Herring Sperm DNA)*, SM1443 *cha-1(p1182); pxEx(cha-1 + htz-1pro::CFP::LacI + PHA-4::YFP +myo-2 mutP + lacO + Herring Sperm DNA),* SM1444 *cha-1(p1182); pxEx(cha-1 + htz-1pro::CFP::LacI + PHA-4::YFP +myo-2 mutP + lacO + Herring Sperm DNA)*, SM1432 *cha-1(p1182); pxEx(cha-1 + htz-1pro::CFP::LacI + PHA-4::YFP + pax-1proWT + lacO + Herring Sperm DNA)*, SM1434 *cha-1(p1182); pxEx(cha-1 + htz-1pro::CFP::LacI + PHA-4::YFP + pax-1proWT + lacO + Herring Sperm DNA)*, SM1463 *cha-1(p1182); pxEx(cha-1 + htz-1pro::CFP::LacI + PHA-4::YFP + pax-1 mutP + lacO + Herring Sperm DNA)*, SM1628 *cha-1(p1182); pxEx(cha-1 + his-24pro::CFP::LacI + PHA-4::citrineYFP + pax-1Delta6proMut + lacO + Herring Sperm DNA),* SM1564 *cha-1(p1182); pxEx(cha-1 + his-24pro::CFP::LacI + PHA-4::citrineYFP + pax-1 mutA + lacO + Herring Sperm DNA),* SM1634 *cha-1(p1182); pxEx(cha-1 + his-24pro::CFP::LacI + PHA-4::citrineYFP + pax-1 mutA + lacO + Herring Sperm DNA),* SM1523 *cha-1(p1182); pxEx(cha-1 + his-24pro::CFP::LacI + PHA-4::citrineYFP + 3X low affinity pha-4 site + lacO + Herring Sperm DNA).* SM1876 *cha-1(p1182); stIs10389 (pha-4::gfp::3xFLAG); pxEx(cha-1 + htz-1pro::mCherry::LacI + M05B5.2 + lacO + Salmon testes DNA).* Transgenic worms used for the Nuclear Spot Assay (NSA) were grown at 24°C on an *E. coli* OP50 lawn or on RNAi plates (see below).

### DNA constructs

For the *pax-1::GFP* cytoplasmic translational fusion construct (BSEM74): a 4.6 kb genomic SacI DNA fragment was cloned from K07C11.1 into pBluescriptIISK+. The resulting plasmid was digested with NsiI, which is located within the *pax-1* locus, and XbaI from the polylinker, to generate a 3 kb *pax-1* fragment that was inserted into PstI/XbaI-digested pPD95.77 (A gift from Dr. Andrew Fire). The resulting *pax-1::GFP* reporter contained approximately 2.4 KB upstream sequences, with GFP fused to *pax-1* within the second predicted exon of *pax-1*. The transcriptional *pax-1* nuclear construct (BSEM274) was made starting with the cytoplasmically-expressed *pax-1::GFP* translational construct, we created a transcriptional fusion by removing all coding sequence. We performed inverse PCR using primers containing BglII tails that flanked the region to be deleted. The linear PCR product was then digested with BglII and re-ligated, placing the GFP translational start site at the same position as the one removed for *pax-1*. PCR products for injection were generated using *pax-1* 5′ as the forward primer and *pax-1* 3′ as the reverse primer.

The transcriptional fusion construct was modified for use in the scanning mutagenesis. A 1.2 kB Bst1071I fragment was removed from BSEM274, and replaced with a 1.9 kb Bst1107I/ApaI fragment from pAP.10 that extends from within the GFP coding region through the *unc-54* poly A addition site was removed. This generates a *pax-1::GFP* transcriptional fusion with the coding sequence for histone H2B fused to the 3′ end of GFP. 276, 277,279,280.

*pha-4::citrineYFP* for the nuclear spot assay was created using QuickChange site directed mutagenesis (Stratagene, #200519). Two mutations, V68L and Q69M (5′-GTT-CAA-3′ mutated to 5′-CTT-ATG-3′), were introduced into the *YFP* sequence of the *pha-4::YFP* (SEM962) [11] to convert *YFP* into *citrineYFP* [91]. The following primers were used: *YFP FW Citrine* 5′-GTCAC TACTTTCGGTTATGGTCTTATGTGCTTCGCCAGATACCCAGATC-3′ and *YFP RV Citrine* 5′GATCTGGGTATCTGGCGAAGCACATAAGACCATAACCGAAAGT AGTGAC-3′.

3X low affinity PHA-4 binding site oligos were designed as in [18] but without restriction sites flanking the 3 tandem binding sites and were created to have an overhang to facilitate repeat formation in the array. The oligos are: Top 5′-CTACTATTTGTCCCTACTATTTGTCCCTACTATTTGTCC-3′ Bottom 5′ GGGACAAATAGTAGGGACAAATAGTAGGGACAAATAGTA-3′ (underlined are the PHA-4 binding sites). Oligos were diluted to a concentration of 2 mg/ml and heated to 95°C for 3 minutes. The temperature was dropped 0.1^0^/sec until 20°C was reached to hybridize the oligos.

### Injections for *pax-1* promoter analysis

Reporter constructs were injected into the germ line of hermaphrodites and stable F2 transgenic roller lines examined. All reporter constructs were injected at 0.5 ng/ml as PCR fragments. Low concentrations of PCR products circumvented artificial expression in pharyngeal cells that has been observed with plasmid constructs [92]. We used pRF4 (*rol-6(su1006)*) as a co-injection marker [93]. The injection mix also included complex DNA (salmon sperm DNA, 1 kb ladder) up to 100 ng/ml, to prevent silencing [59].

### Antibody stains

Antibody staining was performed as described previously [11]. The primary antibodies used were anti-GFP rabbit IgG fraction at 1∶1000 (Molecular Probes), anti-PHA-4 PAb at 1∶1000 [94], the anti-intermediate filament (a-IF) at 1∶3 that recognizes pharyngeal marginal cells [95], and the monoclonal antibody J126, from Dr. Susan Strome, was used at 1∶30 to detect intestinal cells.

### Scanning mutagenesis

All constructs for the scanning mutagenesis were constructed using an inverse PCR strategy. For each mutant, we used a specific pair of primers that flank the 10 bp region to be altered. These primers each carry 5′ tails that contain a restriction site (PstI or ClaI). Following inverse PCR (BSEM279 as template), the linear PCR product was digested with the appropriate restriction enzyme (PstI or ClaI) and re-ligated. Each resulting reporter plasmid contains the restriction site plus a variable number of base pairs in place of the 10 bp of wild-type sequence. For injection, PCR products were generated from each mutant plasmid. All constructs were sequenced to confirm the predicted sequence.

### Nuclear spot assay

Transgenic lines for the Nuclear Spot Assay were as follows: no target control (SM1560), 3X low affinity *pha-4* binding sites (SM1523), *myo-2* wild-type promoter (SM1476, SM1429) bearing two high affinity PHA-4 binding sites [2], *myo-2* promoter bearing mutagenized FoxA sites (SM1443, SM1444) [2], *pax-1* wild-type promoter (SM1432, SM1434), *pax-1* promoter with a mutagenized FoxA site (SM1463, SM1628) and *pax-1* promoter with a mutagenized positive regulator site (SM1564, SM1634) (see below). SM1560 was created by injecting *cha-1(p1182)* worms with Xho1-linearized *pha-4*::citrine*yfp* plasmid (bSEM1045) (1 ng/µl), *his24promoter::CFP::LacI* PCR product [63] (2.5 ng/µl), a 10 kb Sph1/Kpn1 fragment from lacO multimeric plasmid pSV2-dhfr-8.23 (3 ng/µl) [96], *cha-1* plasmid (RM527P, a gift from J. Rand) linearized with Apa1 (2 ng/µl) for rescue, and sheared herring sperm DNA to make 100 ng/µl total DNA. For SM1476 and SM1429, 499 bp of the endogenous *myo-2* promoter upstream of the start codon was used in addition to the components listed for SM1560 with one difference, *CFP::lacI* expression was driven by the *htz-1* promoter (BSEM995) [63]. SM1443 and SM1444 were created similar to SM1476 and SM1429 but with a *myo-2* promoter bearing two mutated PHA-4 binding sites [2]. For SM1434 a 240 (bp) fragment of the *pax-1* promoter upstream of the start codon was used (the fragment contains one PHA-4 binding site (TGTTTGC)). SM1463 carried an altered version of the 240 (bp) *pax-1* promoter in which the PHA-4 binding site was mutated from TGTTTGC to ATCGATT (*MutP*). Both SM1463 and SM1434 were injected with *htz-1pro::CFP::LacI*. For SM1564 and SM1634 a positive regulator site −40 to −50 upstream of the TSS was mutated from TTGAGATTAA to CAATCGATTG. SM1876 was created by injecting SM1754 *cha-1(p1182); stIs10389 (pha-4::gfp::3xFLAG)* worms with *cha-1,* a 440 (bp) M05B5.2 promoter fragment [2], and *htz-1pro::mCherry::LacI* that has a premature stop codon at the end of the mCherry sequence, thus failing to make any mCherry::LacI. SM1876 was used to examine whether decompaction reflected an artificial interaction between LacI and PHA-4. Nuclear spot assays were performed as described previously [11], [50], [51], [63], with the following modifications: sequential scan images were acquired using the Andor Revolution XD microscopy system (≤16E stages). For later stages, images were acquired using an Olympus FluoView FV1000 confocal microscope (for *pax-1 mutA*) or a Leica DM RXE confocal (for everything else).

To determine copy number, worms were grown at restrictive temperature for *cha-1(p1182*) (25°), and treated with bleach to synchronize embryos. Four 10 cm OP50 plates of moving, nonCha-1 L3 animals were harvested for DNA isolation by phenol chloroform extraction and ethanol precipitation. qPCR was performed for promoter regions and normalized to *act-1* using a LightCycler PCR machine with LightCycler FastStart DNA Master^Plus^ SYBR Green 1 kit (Roche) for quantitation. qPCR indicated a copy number of ≤200 for each promoter.

### Heat shock

Gravid mothers were dissected and embryos collected in a PCR tube. Heat-shock was administered in a PCR machine. Embryos were initially incubated at 20°C for 75 min. After the initial incubation, the temperature was raised gradually to 33°C at a rate of 0.1°C/second. Embryos were then incubated at 33°C for 30 minutes. Following heat shock, the temperature was gradually lowered to 20°C at a rate of 0.1°C/second, and embryos incubated at 20°C for 5 hours.

### Image analysis

Perkin Elmer Volocity was used to calibrate images for true X and Y pixel dimensions to ensure accurate spatial measurements. Classifiers were designed to select CFP::LacI areas and PHA-4::YFP^+^ cells using an intensity threshold. The CFP::LacI classifier included separation of touching objects, removal of noise and exclusion of objects smaller than 0.25 micron^2^. The PHA-4::YFP classifier was modified to remove noise. CFP::LacI areas within PHA-4::YFP^+^ cells were considered “inside the pharynx” and the remainder as “outside the pharynx.” Proofreading of selections was performed blind by comparing measurements with images. Area measurements (INT Area (micron^2^)*10) were analyzed using Cox regression models to evaluate differences in chromosome area with location (inside the pharynx versus outside), developmental stage, or transgenic line. While Cox regression models were originally developed for analyzing survival data, their semi-parametric nature made them suitable for analyzing data following a non-standard distribution that was difficult to capture parametrically.

### RNA interference screen and analysis of general decompaction and PHA-4 binding

RNAi by bacterial feeding was performed similarly to [15]. HT115 bacteria [97] expressing dsRNA for *gfp, spr-5, rbr-2, npp-11, emr-1, nhr-60, ima-3, lem-3, zyg-12, lmn-1, lin-49, lin-59, set-17, set-16, set-2, set-1, met-2, hda-3, hda-4, tsn-1, prg-1, sago-2, prg-2, csr-1, top-1, ergo-1, chd-3, tam-1, lin-35, or hil-7* were grown in liquid cultures for 8 hours and seeded onto plates containing 8 mM IPTG (Sigma) and 50 g/ml Carbenicillin (Sigma). All RNAi clones were derived from the Ahringer library [98]. The emr-1 clone was validated by sequencing using pPD129_for 5′-GAGTGAGCTGATACCGCTCG-3′ and pPD129_rev 5′-CACGACGGTGTATTTCGACGGC-3′ primers at the Dana-Farber/Harvard Cancer Center DNA Resource Core. Adult SM1634 worms were bleached and ∼50 embryos were placed on RNAi plates (Po). For every experiment, F1 progeny embryos from 5 6 cm plates were collected by bleaching and analyzed, and each experiment was repeated at least twice. Images were acquired from live embryos using an Olympus FluoView FV1000 confocal microscope and DeltaVision RT Deconvolution System and SoftWoRx software (Applied Precision). A multitrack setting was used to acquire separate CFP and YFP images from slices through the pharynges of comma to 1.5fold-stage embryos. Embryos were scored for general non-tissue specific extrachromosomal array decompaction and for the number of nuclei containing PHA-4-::YFP colocalized with CFP::LacI.

## Supporting Information

Figure S1 Lineage of *pax-1*::GFP^+^ pharyngeal cells. (A) Cell nuclei positions in the pharynx (Adapted from [100]). Highlighted are the twelve nuclei that express *pax-1::GFP.* (B) the Lineage of *pax-1*::GFP^+^ pharyngeal cells. Eleven of these express *pax-1::GFP*, and the 12^th^ (e1VR) may as well, although this has not been confirmed unambiguously.
(10.21 MB TIF)

Figure S2 Deletion analysis of the *pax-1* promoter. The PAX-1::GFP cytoplasmic expression construct (translational construct) was used for this analysis. Expression is pharyngeal, but identification of individual cells was difficult. Magenta box indicates the PHA-4 binding site (TGTTTGC). Progressively larger deletions from the original 2.5 kb upstream sequence resulted in a gradual loss of GFP intensity, with eventually a complete loss of expression when the predicted PHA-4 site was removed. Images representative of strong, weak and no GFP expression are shown below the schematic.
(5.10 MB TIF)

Figure S3 Ectopic expression of *pax-1MutP*::GFP reporter in epidermal cells (A) and seam cells (B).
(2.30 MB TIF)

Figure S4 Characterization of negative regulatory elements in the *pax-1* promoter. (A) D18 resulted in an increased number of pharyngeal cells expressing the GFP reporter (20 cells), but with no significant non-pharyngeal expression. (B) D20 displayed increased numbers of GFP-expressing cell in the pharynx, as well as non-pharyngeal expression, epidermal expression is shown here.
(1.78 MB TIF)

Figure S5 PHA-4 binding to an additional pharyngeal target. (A) Quantitation of embryos with co-localized CFP::LacI and PHA-4::YFP in two transgenic lines bearing a WT C44H4.1 (1 kb) promoter. (B) Numbers of embryos scored for binding for all transgenic lines in this study. (C) PHA-4::GFP binding to a Nuclear Spot Assay array bearing the promoter of M05B5.2 in a transgenic line that lacks mCherry::LacI. Binding to the array and decompaction is observed as an intense PHA-4::GFP signal (Arrows; Left image) compared to a transgenic line expressing PHA-4::GFP without any target promoter (Right image). (D) PHA-4 binding is maintained on mitotic chromosomes (Arrows) (E) The diameter of pharyngeal nuclei at different developmental stages.
(8.08 MB TIF)

Figure S6 *emr-1* RNAi reduces the expression of EMR-1 in all cells. (A) EMR-1 antibody stain reveals its position at the nuclear periphery in a nuclear spot assay transgenic line (B) EMR-1 signal is lost after RNAi. A secondary antibody against LacI was used as a positive control for antibody staining (LacI alone shown in the inset).
(5.57 MB TIF)

Figure S7 Comparison of area measurements versus volume measurements for array size. (A) Area or (B) Volume of pseudo-chromosomes in the pharynx were measured at the comma and 1.5Fold stage in transgenic lines carrying either a WT *pax-1* promoter or a *MutP pax-1* promoter. Three embryos per stage were analyzed. Each dot on the plot represents a pseudo-chromosome.
(7.06 MB TIF)

Table S1 *pax-1* reporters are activated at the bean stage. (A) GFP expression assayed in two transgenic lines. (A) is a line carrying a transcriptional fusion of *pax-1WTpro*::GFP. Onset of expression was detected at the bean stage. This expression pattern was recapitulated using an integrated PAX-1::GFP translational fusion. (B) Mutations in a predicted PHA-4 binding site (*mutP*) or a second activation site (*mutA*) interfere with activation at any stage. n =  number of embryos.
(2.64 MB TIF)

Table S2 Number of nuclei assayed for pseudo-chromosome size. The numbers are broken down per promoter, developmental stage and for the location of pseudo-chromosomes inside the pharynx versus outside the pharynx.
(6.97 MB TIF)

Table S3 Number of embryos assayed for de-compaction for each RNAi treatment. (A) Colors indicate different categories. (B) the number of embryos assayed for the proportion of bound arrays.
(9.28 MB TIF)
