# Supplementary figures and images for: Dynamic Chromatin Organization during Foregut Development Mediated by the Organ Selector Gene PHA-4/FoxA
Source: PLoS Genet. 2010 Aug 12;6(8):e1001060. doi: 10.1371/journal.pgen.1001060 (PMC2920861; doi:10.1371/journal.pgen.1001060)

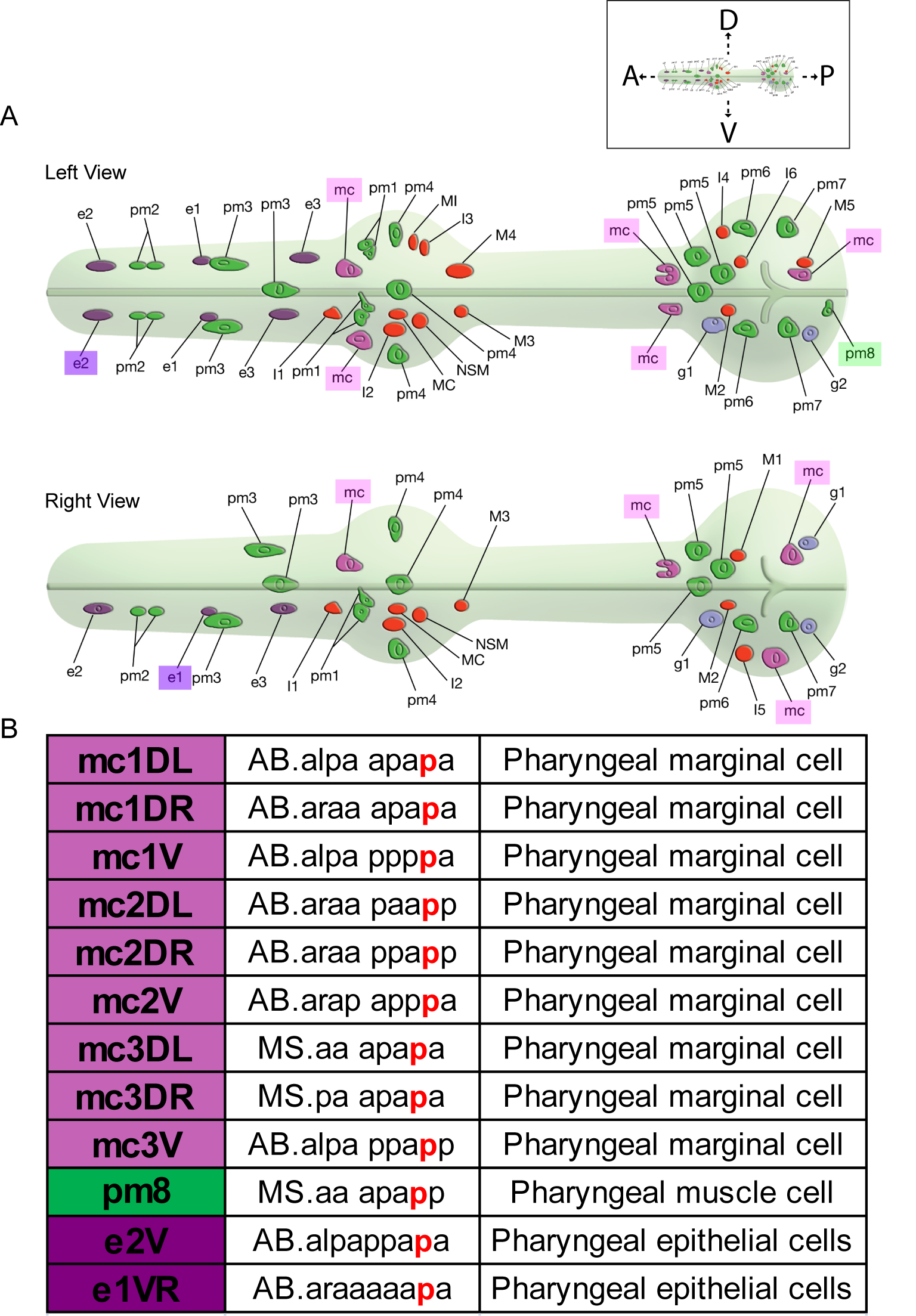

Supplement: Figure S1 — Lineage of pax-1::GFP+ pharyngeal cells. (A) Cell nuclei positions in the pharynx (Adapted from [100]). Highlighted are the twelve nuclei that express pax-1::GFP. (B) the Lineage of pax-1::GFP+ pharyngeal cells. Eleven of these express pax-1::GFP, and the 12th (e1VR) may as well, although this has not been confirmed unambiguously. (10.21 MB TIF) [file pgen.1001060.s001.tif]

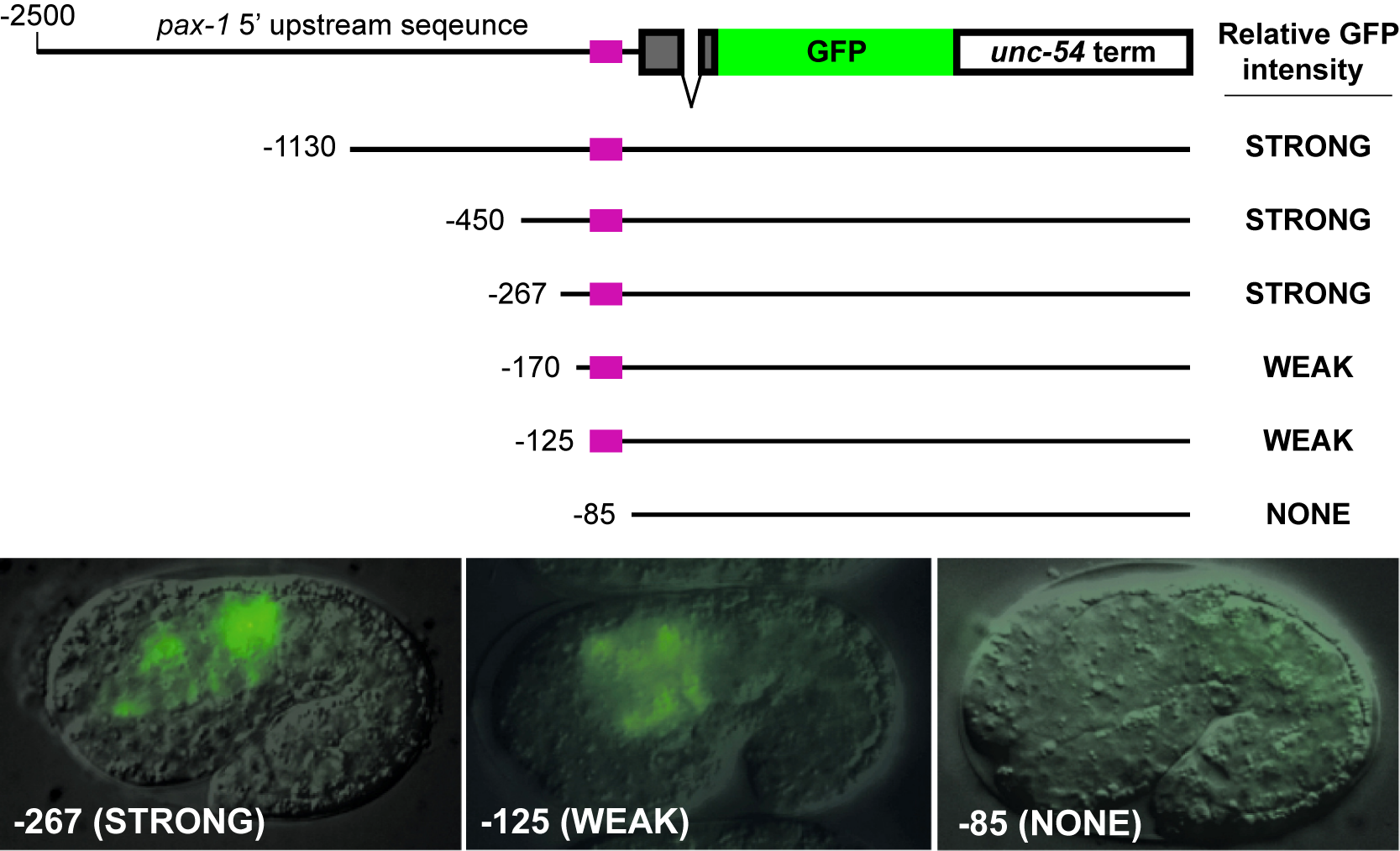

Supplement: Figure S2 — Deletion analysis of the pax-1 promoter. The PAX-1::GFP cytoplasmic expression construct (translational construct) was used for this analysis. Expression is pharyngeal, but identification of individual cells was difficult. Magenta box indicates the PHA-4 binding site (TGTTTGC). Progressively larger deletions from the original 2.5 kb upstream sequence resulted in a gradual loss of GFP intensity, with eventually a complete loss of expression when the predicted PHA-4 site was removed. Images representative of strong, weak and no GFP expression are shown below the schematic. (5.10 MB TIF) [file pgen.1001060.s002.tif]

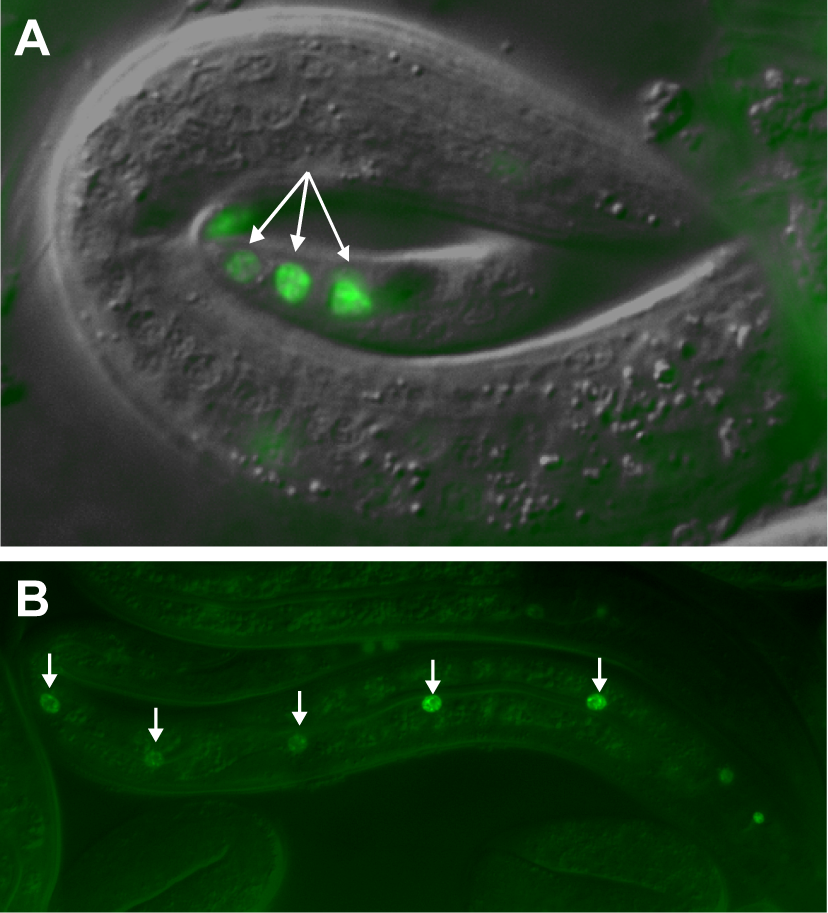

Supplement: Figure S3 — Ectopic expression of pax-1MutP::GFP reporter in epidermal cells (A) and seam cells (B). (2.30 MB TIF) [file pgen.1001060.s003.tif]

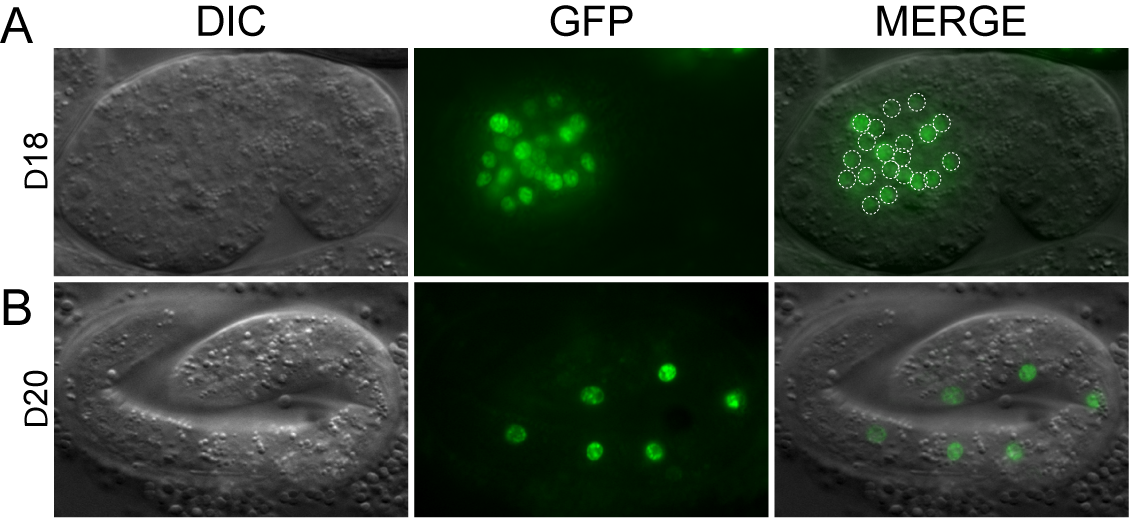

Supplement: Figure S4 — Characterization of negative regulatory elements in the pax-1 promoter. (A) D18 resulted in an increased number of pharyngeal cells expressing the GFP reporter (20 cells), but with no significant non-pharyngeal expression. (B) D20 displayed increased numbers of GFP-expressing cell in the pharynx, as well as non-pharyngeal expression, epidermal expression is shown here. (1.78 MB TIF) [file pgen.1001060.s004.tif]

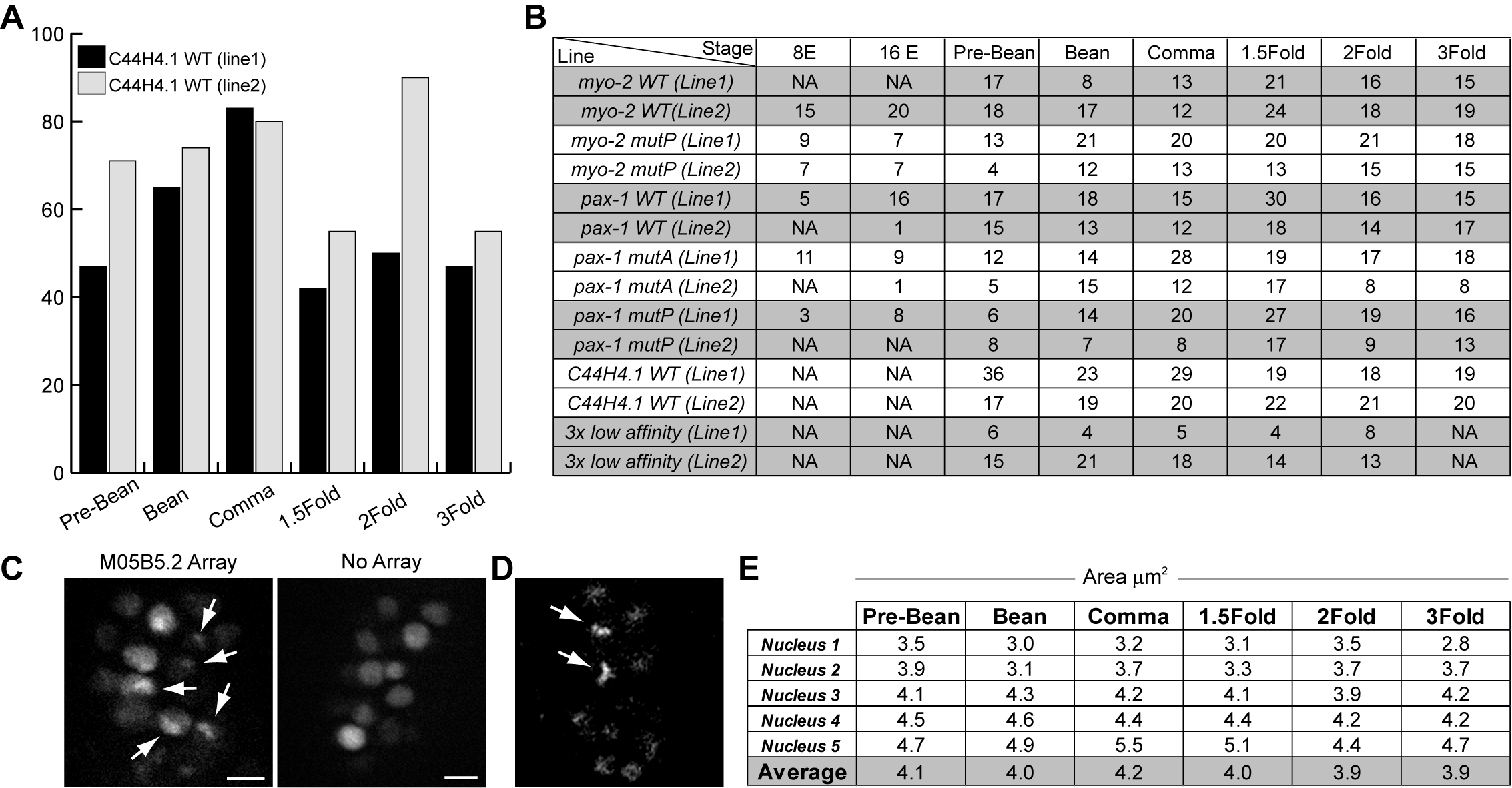

Supplement: Figure S5 — PHA-4 binding to an additional pharyngeal target. (A) Quantitation of embryos with co-localized CFP::LacI and PHA-4::YFP in two transgenic lines bearing a WT C44H4.1 (1 kb) promoter. (B) Numbers of embryos scored for binding for all transgenic lines in this study. (C) PHA-4::GFP binding to a Nuclear Spot Assay array bearing the promoter of M05B5.2 in a transgenic line that lacks mCherry::LacI. Binding to the array and decompaction is observed as an intense PHA-4::GFP signal (Arrows; Left image) compared to a transgenic line expressing PHA-4::GFP without any target promoter (Right image). (D) PHA-4 binding is maintained on mitotic chromosomes (Arrows) (E) The diameter of pharyngeal nuclei at different developmental stages. (8.08 MB TIF) [file pgen.1001060.s005.tif]

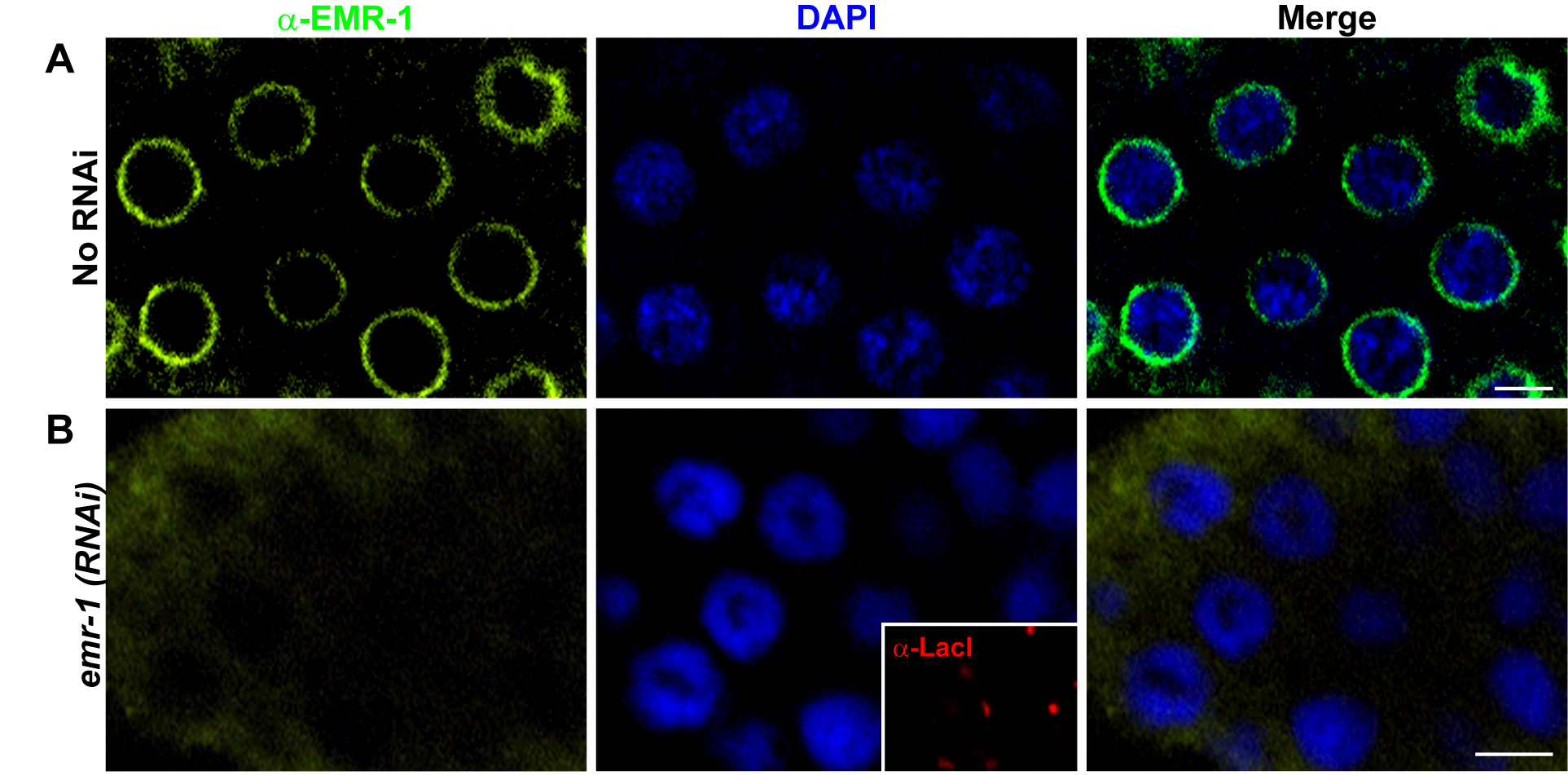

Supplement: Figure S6 — emr-1 RNAi reduces the expression of EMR-1 in all cells. (A) EMR-1 antibody stain reveals its position at the nuclear periphery in a nuclear spot assay transgenic line (B) EMR-1 signal is lost after RNAi. A secondary antibody against LacI was used as a positive control for antibody staining (LacI alone shown in the inset). (5.57 MB TIF) [file pgen.1001060.s006.tif]

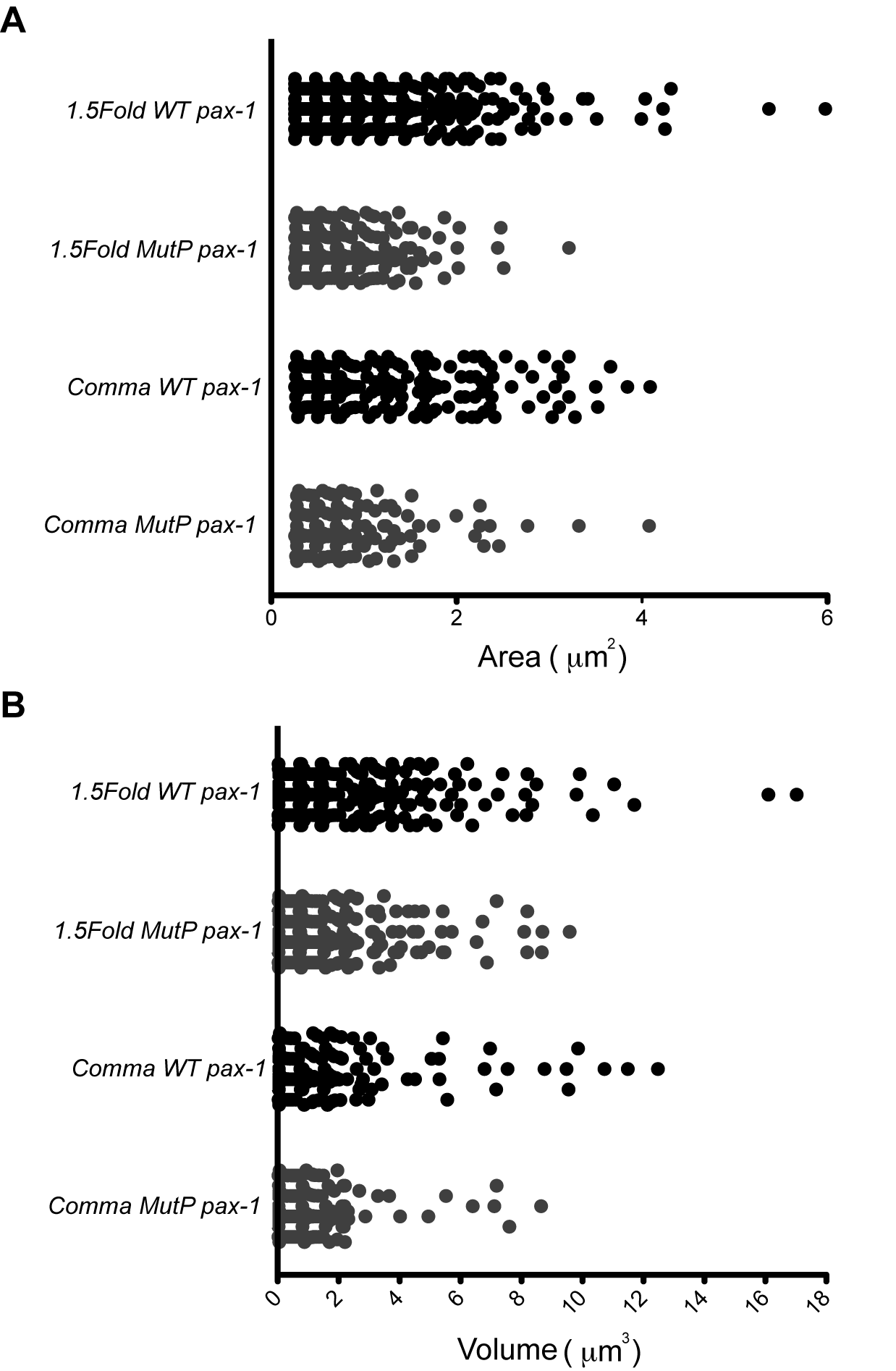

Supplement: Figure S7 — Comparison of area measurements versus volume measurements for array size. (A) Area or (B) Volume of pseudo-chromosomes in the pharynx were measured at the comma and 1.5Fold stage in transgenic lines carrying either a WT pax-1 promoter or a MutP pax-1 promoter. Three embryos per stage were analyzed. Each dot on the plot represents a pseudo-chromosome. (7.06 MB TIF) [file pgen.1001060.s007.tif]

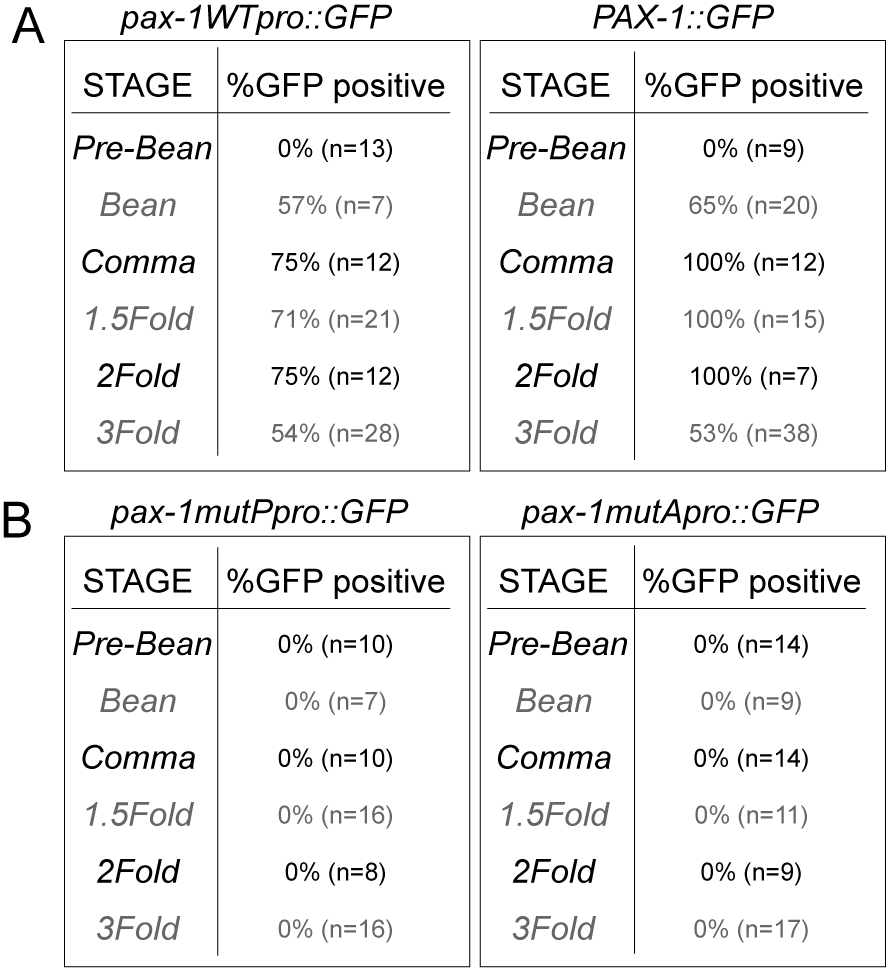

Supplement: Table S1 — pax-1 reporters are activated at the bean stage. (A) GFP expression assayed in two transgenic lines. (A) is a line carrying a transcriptional fusion of pax-1WTpro::GFP. Onset of expression was detected at the bean stage. This expression pattern was recapitulated using an integrated PAX-1::GFP translational fusion. (B) Mutations in a predicted PHA-4 binding site (mutP) or a second activation site (mutA) interfere with activation at any stage. n = number of embryos. (2.64 MB TIF) [file pgen.1001060.s008.tif]

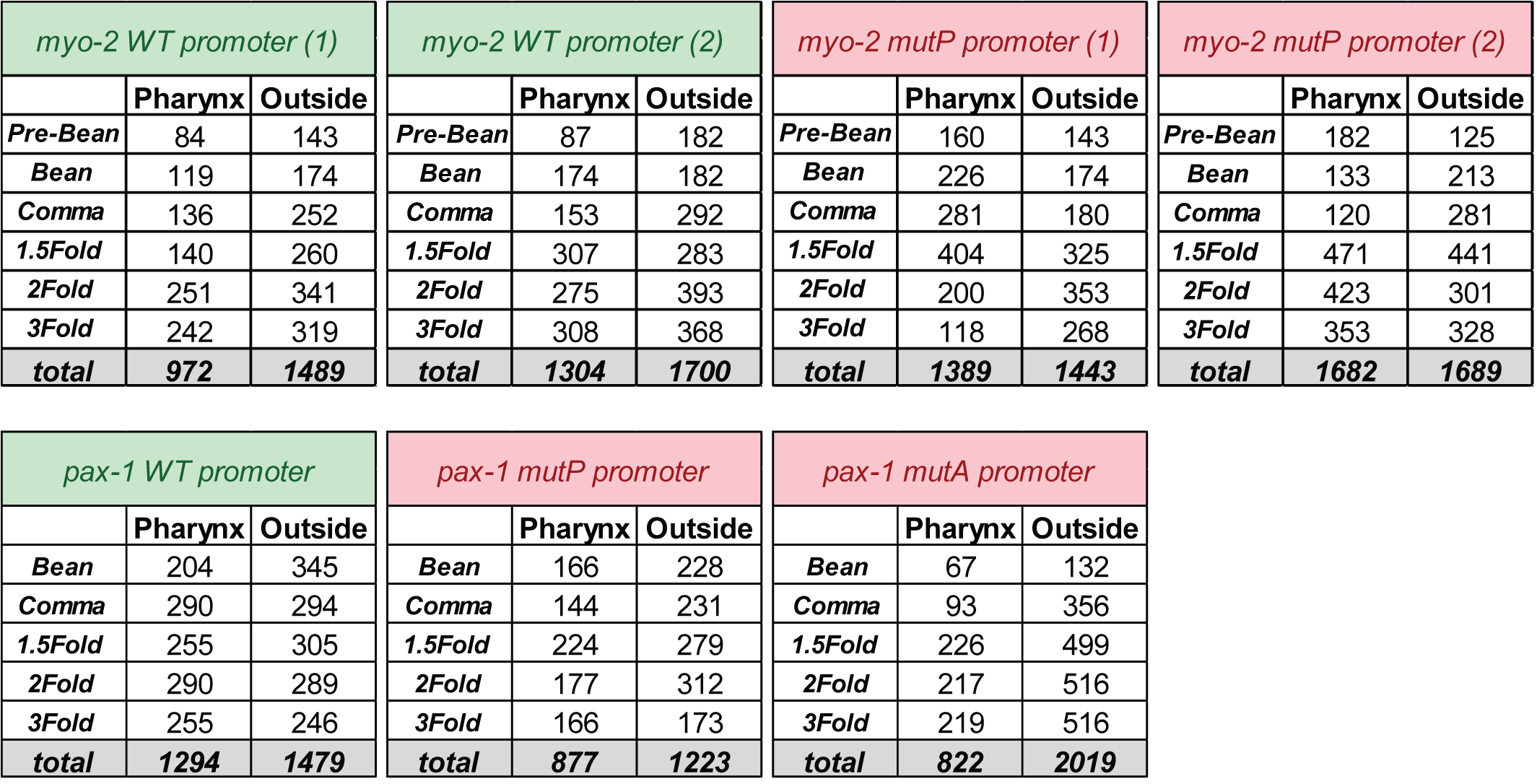

Supplement: Table S2 — Number of nuclei assayed for pseudo-chromosome size. The numbers are broken down per promoter, developmental stage and for the location of pseudo-chromosomes inside the pharynx versus outside the pharynx. (6.97 MB TIF) [file pgen.1001060.s009.tif]

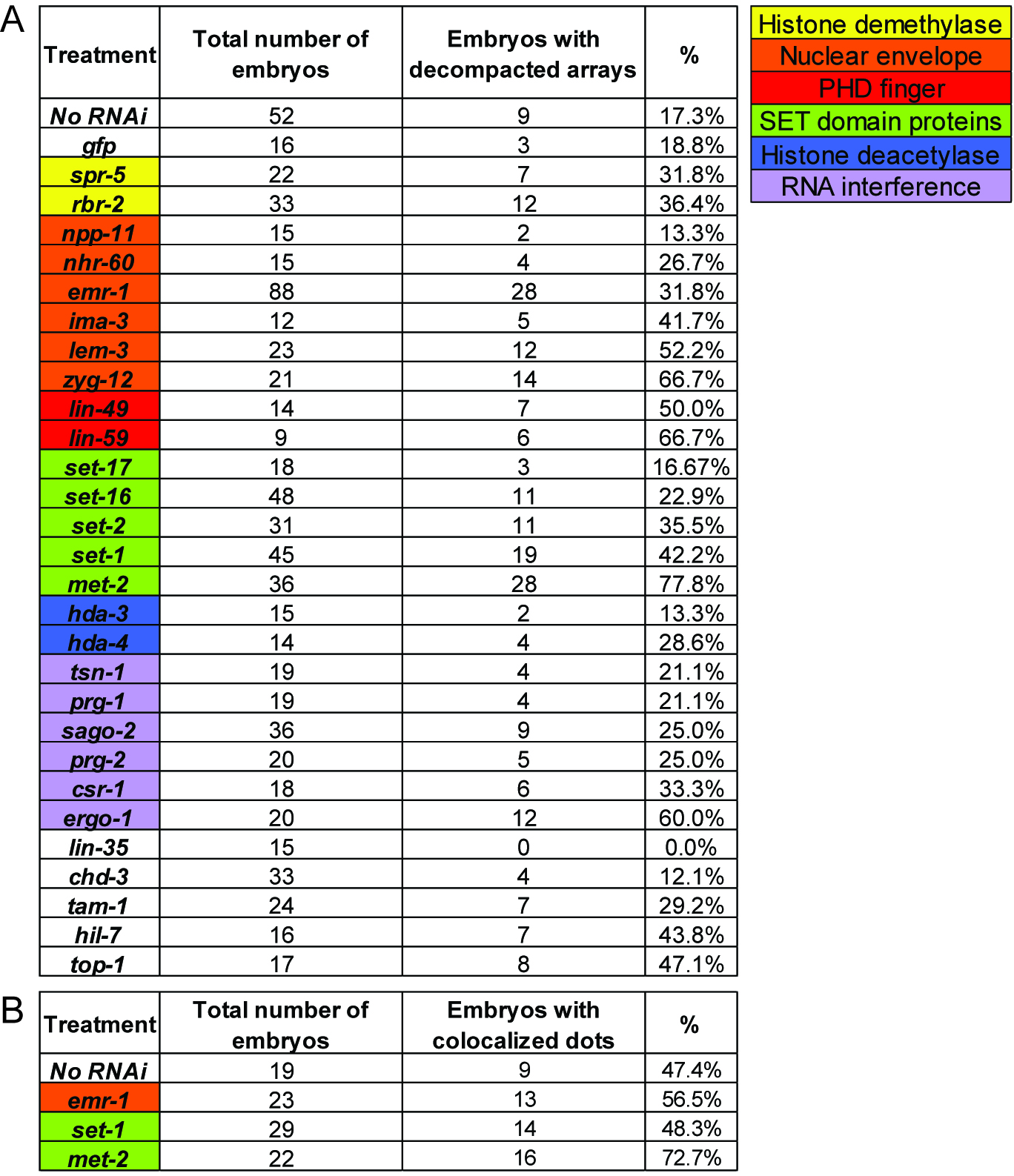

Supplement: Table S3 — Number of embryos assayed for de-compaction for each RNAi treatment. (A) Colors indicate different categories. (B) the number of embryos assayed for the proportion of bound arrays. (9.28 MB TIF) [file pgen.1001060.s010.tif]
